# Advances in Flexible Organic Photodetectors: Materials and Applications

**DOI:** 10.3390/nano12213775

**Published:** 2022-10-26

**Authors:** Hossein Anabestani, Seyedfakhreddin Nabavi, Sharmistha Bhadra

**Affiliations:** Department of Electrical and Computer Engineering, McGill University, Montreal, QC H3A 0E9, Canada

**Keywords:** photodetectors, organic materials, flexible, hybrid material systems, wearable electronics

## Abstract

Future electronics will need to be mechanically flexible and stretchable in order to enable the development of lightweight and conformal applications. In contrast, photodetectors, an integral component of electronic devices, remain rigid, which prevents their integration into everyday life applications. In recent years, significant efforts have been made to overcome the limitations of conventional rigid photodetectors, particularly their low mechanical deformability. One of the most promising routes toward facilitating the fabrication of flexible photodetectors is to replace conventional optoelectronic materials with nanomaterials or organic materials that are intrinsically flexible. Compared with other functional materials, organic polymers and molecules have attracted more attention for photodetection applications due to their excellent photodetection performance, cost-effective solution-fabrication capability, flexible design, and adaptable manufacturing processes. This article comprehensively discusses recent advances in flexible organic photodetectors in terms of optoelectronic, mechanical properties, and hybridization with other material classes. Furthermore, flexible organic photodetector applications in health-monitoring sensors, X-ray detection, and imager devices have been surveyed.

## 1. Introduction

Photodetectors (PDs) are critical components for various electronic applications such as image sensors, medical monitoring, optical communication, and manufacturing process monitoring. On the other hand, over the past decade, many efforts have been dedicated to the fabrication of flexible devices such as sensors [1,2], memory devices [3], power modules, and wearable electronics [4,5]. Incorporating optoelectronic functions into flexible [6] and soft surfaces, particularly human skin, is at the forefront of multidisciplinary research. For example, lightweight and conformal image sensors with different wavelength sensitivity will open up numerous applications in wearable electronics, robotics, and the automotive industry. Due to the rapid rise of the Internet of Things (IoT), there is an increasing demand for smart and unobtrusive PDs that can be adhered to curved surfaces or applied to the body (either via the skin or implanted). These devices are able to revolutionize the healthcare industry by enabling accurate, continuous detection of physiological signals without interfering with human daily activities. In recent years, several promising approaches have been utilized for flexible photodetector fabrication, such as the application of silicon devices incorporated by flexible interconnects on a patterned polymer substrate [7] and conformal thin-film devices adhering to malleable biological organs [8], skin [9], and textiles [10] via manipulation of inorganic PDs. However, the above approaches do not completely satisfy the requirements of flexible PDs. For example, in the case of silicon devices, the thickness must be reduced to tens or hundreds of nanometers, which reduces light absorption significantly. In addition, due to their stiffness, inorganic flexible devices fail to conform to curved surfaces.

An alternative approach involves the application of solution-processable semiconductor materials, including multi-conjugated organic semiconductors, colloidal quantum dots (QDs) [11,12,13], perovskites [14,15,16], and two-dimensional materials [17,18]. In contrast to bulk materials, nanoscale materials (such as thin films with a thickness of a few micrometers) exhibit a much lower flexural rigidity [19]. Moreover, these nanomaterials and organic materials exhibit excellent optoelectronic properties, such as high photo-absorption coefficients or narrow-bandwidth light emission. There has been a great deal of progress reported with solution-processed PDs based on QDs and perovskites. However, toxic materials are used in these PDs for wearable electronics, which remain a cause for concern [20]. Additionally, PDs based on two-dimensional materials are limited in spectral response, response time, air stability, and fabrication for large areas [21]. Unlike other solution-processed materials, organic semiconductors (molecules and polymers) exhibit low toxicity, broad spectral response, relatively fast response times, and the ability to be deposited on soft, flexible substrates, including plastics and elastomers, using blade coating, roll-to-roll printing, or inkjet printing for large areas [22].

Recent developments in organic heterojunction structures have enhanced the charge photogeneration efficiency of organic thin films, achieving detectivity up to 10^14^ Jones and a photovoltaic efficiency equal to inorganic PDs under sunlight [23]. In addition to this, the absorption wavelength of organic photodetectors (OPDs) can be easily tuned by modifying the chemical structure of the active material [24], allowing a tunable spectral range including ultraviolet–visible (UV–vis), near-infrared (NIR), and even X-ray wavelengths. Several review articles have focused on OPDs, mainly analyzing the performance, figure of merits, and working principles of OPDs on glass substrates [22,25], and not the ones on flexible substrates. This paper presents an overview of recent advances in flexible OPDs. We begin with a summary of the fundamental operating principles of OPDs, a description of the various types of OPDs developed to date, as well as the key figure of merits relevant to the flexible applications. Next, we discuss various novel materials for active layers, including organic materials and their hybrids with other material systems developed to enhance the light-sensing performance and mechanical properties of OPDs. Finally, we explore applications enabled by flexible OPDs, such as flexible health-monitoring sensors, flexible image sensors, and X-ray detectors.

## 2. Flexible OPD Background

### 2.1. Flexible OPD Working Principle

In organic semiconductors, low dielectric constants (ɛ_r_ = 3–4) and the localized nature of the excited states give rise to coulombically bound electron–hole pairs called excitons. Typical binding energies of excitons in organic materials have been reported to range between 0.3 and 0.5 eV [26]; therefore, large photocurrents require efficient separation of excitons.

**Figure 1:**
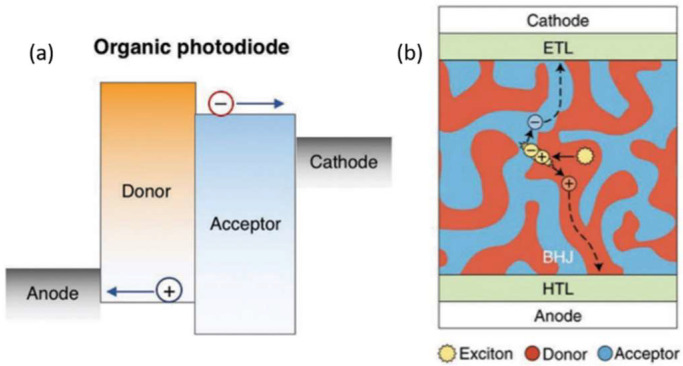
Organic photodetectors. (**a**) Band diagram of an organic photodiode depicting charge separation of excitons at donor–acceptor interface. (**b**) Use of bulk heterojunction (BHJ) structure with nanoscale domains enables optimal harvesting excitons. Reprinted with permission from [20] ©2021 Wiley-VCH GmbH.

An effective way to separate electron–hole pairs in organic materials is to form a heterojunction between electron-donating (donor) and electron-accepting (acceptor) materials, where the differing electron affinity and/or ionization potentials provide a driving force for charge separation and send them towards their corresponding electrode, which has been shown in Figure 1a.

Robust harvest of excitons without compromising light absorption is enabled by the use of a bulk heterojunction (BHJ) structure [27,28]; the structure of a BHJ is shown in Figure 1b. A BHJ consists of a bicontinuous interpenetrating network of the donor and acceptor materials with nanoscale phase segregation. The large interfacial area significantly reduces the distance required for the excitons to travel before reaching an interface where they can separate into free charges. A photocurrent is created when the charge is successfully extracted at the electrodes without undergoing recombination [29]. Organic semiconductors exhibit low charge mobilities compared to their inorganic counterparts, ranging from 10^−6^ to 10^−3^ cm^2^ V^−1^ s^−1^ to around 10 cm^2^ V^−1^ s^−1^ in amorphous or disordered films [30]. Electron–hole pairs can also form intermolecular charge-transfer states at the heterojunction, typically with energy below the optical bandgap [31]. These charge-transfer states are weakly optically coupled to the ground state and may lead to a broadening of the absorption edge, as reported for a wide range of systems [32]. Most OPDs developed to date are based on a donor–acceptor heterojunction, which can efficiently separate photogenerated electrons and holes. While some devices employ a planar heterojunction (bilayer) structure [33], most have a BHJ structure. Many of the state-of-the-art OPDs and organic solar cells are based on very similar material systems. This is most likely due to these material systems’ high charge generation yields. However, it is important to point out that systems with optimal solar cell efficiencies may not necessarily be the best systems for photodetection. For instance, while materials in organic solar cells are designed to maximize the absorption range across the solar spectrum, PDs used for wearable applications have a very different set of requirements regarding the ideal absorption range. Therefore, there exists a large amount of room for improvements in terms of material and device designs for optimizing the performance of OPDs for applications other than solar energy.

The three most common configurations of today’s OPDs are organic photodiodes, photoconductors, and phototransistors. The general configurations, working mechanisms, and performance characteristics of OPDs are discussed in this section.

### 2.2. Flexible OPD Configuration

#### 2.2.1. Organic Photodiodes

In vertical two-terminal organic photodiodes (Figure 1a), there is an anode, a hole-transporting layer (HTL), an organic photoactive layer, an electron-transporting layer (ETL), and a cathode. Organic photoactive layers are typically composed of an organic BHJ, a mixture of organic electron-donating and electron-accepting materials. Four main processes are involved in photocurrent generation in the BHJ: exciton generation, exciton dissociation, charge transport, and charge collection. The use of the HTL and ETL in an OPD facilitates charge extraction at the BHJ/electrode interfaces, resulting in photocurrent generation. A photodiode’s photoresponse is based on collecting photogenerated charge carriers, allowing it to operate either self-powered or with a reverse bias [20].

#### 2.2.2. Organic Photoconductors

Organic photoconductors (OPCs) are horizontal two-terminal devices with two symmetrical electrode contacts, an organic photoactive layer, and a substrate (Figure 1b). OPCs are typically operated with an external bias, and photodetection is defined as the detection of light absorption by measuring the change in electrical conductivity of the photoactive layer [20].

#### 2.2.3. Organic Phototransistors

The organic phototransistors (OPTs) are three-terminal devices, having an organic photoactive channel layer, a dielectric layer, and three electrodes, including the gate, drain, and source electrodes (Figure 1). In OPTs, one type of charge carrier in the channel layer is conducted from the drain electrode and the source electrode, whereas the gate electrode traps the other type of charge carrier. The carrier concentration in the organic layer increases with the photogenerated charge carriers, thus contributing to the change in drain-source current (I_DS_). The advantage of OPTs is their high gain due to the photogate effect. As a result, a single photon incident on the OPT can induce a large number of charge carriers in the channel layer, resulting in a large photocurrent [20].

### 2.3. Figure of Merits

#### 2.3.1. Optoelectronic Performance

##### EQE and Responsivity

External quantum efficiency (*EQE*) is a dimensionless parameter that is defined as the ratio of the number of charge carriers circling across the PDs to the number of incident photons. Responsivity (*R*) is defined as the ratio of photocurrent measured for the PDs to incident light power with a unit of AW^−1^. In this case, *EQE* and *R* represent the conversion efficiency of an optical device to an electric device. *EQE* and *R* are defined as [22]:

(1) $$\;R=\frac{I_{{}_{ph}}}{P}\;$$

(2) $$EQE=\frac{Rhv\;}{q}$$

where *I_ph_* is the photocurrent measured for PDs, *P* is the incident light power, *h* is Planck’s constant, *υ* is the light-wave frequency, and *q* is the elementary charge, respectively. It is worth mentioning that the EQE of photodiode-type OPDs is limited to 100%, whereas the EQE of OPTs and photo multiplication (PM)-type OPDs can exceed 100%. Enhanced photoresponses are possible with light-triggered charge injection in OPTs and PM-type OPDs.

##### Response Time

The rise time (r) of a PD is defined as the time interval for the photocurrent of the PD to rise from 10% to 90% of its maximum value upon the arrival of light, and the fall time (f) is defined as the time interval for the photocurrent of the PD to drop from 90% to 10% of its maximum value upon the removal of light.

##### Noise

OPD noise (*S_n_*) is closely related to shot noise (*S_shot_*), thermal noise (*S_thermal_*), and 1/f noise (*S_1/f_*), which can be calculated as follows [22]:

(3) $$S_{n}=\sqrt{S_{shot}^{2}+S_{thermal}^{2}+S_{\frac{1}{f}}^{2}\;}\;$$

(4) $$S_{\mathrm{shot}}\;=\;\sqrt{2q\times I_{d}\times B}$$

(5) $$S_{thermal}=\sqrt{\frac{4kTB}{R_{sh}}}$$

where *I_d_* is the PDs’ dark current, *B* is the detection bandwidth, *k* is the Boltzmann constant, *T* is the absolute temperature, and *R_sh_* is the PDs’ shunt resistance. PDs suffer from *S_shot_* due to the electric charges that contribute to the statistical fluctuation of device current. The thermal activation of the charge carriers in the PDs causes *S_thermal_*, also known as Nyquist noise or Johnson noise. The white noise is defined as the sum of *S_shot_* and *S_thermal_*, which is independent of the detection frequency. The *S_1/f_*, also known as flicker noise, varies with frequency and is most prominent in the low-frequency range. The trapping/de-trapping of charge carriers during the charge generation–recombination process is one of the sources of *S_1/f_*.

##### Specific Detectivity

Specific detectivity (D*) is calculated by [22]:

(6) $$D^{*}=\frac{\sqrt{A.B}\;}{NEP}=\frac{R\sqrt{A}}{S_{n}}\;$$

where *A* is the active area of the PD, *NEP* is the noise equivalent power, and *R* is a sensitivity measure indicating the power level of incident light that produces a signal-to-noise ratio of one in a 1 Hz output bandwidth, respectively. The precise determination of *R* and *S_n_* is essential for calculating D*. In OPDs, the *S_shot_* is typically regarded as the primary noise source and is used to calculate D*. However, the measured *S_n_* can be significantly greater than the *S_shot_*. This can result in an overestimated D* due to thermal noise, particularly in OPDs with photoactive layers of narrow-bandgap materials. Due to the frequency dependence of the photoresponse and the *S_n_*, the D* decays in the *S_1/f_*-limited low-frequency range and the response speed-limited high-frequency range. To calculate the D*, both the frequency-dependent *R* and *S_n_* should be considered.

#### 2.3.2. Stability and Durability

Although OPDs fabricated on thin plastic foil exhibit excellent mechanical compliance and durability, it remains difficult to achieve high performance, mechanical durability, and stability in air and water simultaneously. This is due to the reduced thickness of the plastic substrate and encapsulation layer, which results in an insufficient barrier to moisture and oxygen for wearable device applications [34]. While rigid OPDs can easily achieve long device lifetimes by encapsulation with epoxy and glass [35], new strategies for effectively protecting the active layer without introducing mechanical stiffness are required for wearable devices.

Jinno et al. [10] created the first washable and stretchable organic photodiodes by encapsulating the free-standing ultrathin organic photodiode between two pre-stretched 500-micrometer-thick acrylic elastomers. The organic photodiode achieved high solar cell efficiency (7.9%) and stretch ability (52%), even when completely submerged in water, using this method. Furthermore, it was discovered that after compression for 20 cycles with 100 min of water exposure, 80 percent of the initial photovoltaic efficiency was maintained.

## 3. Flexible OPDs Advances

Using flexible organic semiconductors, flexible OPDs can be designed for flexible applications on curved, conformable, and foldable substrates. Flexible OPD fabrication also necessitates using flexible substrates, flexible transparent conductive electrodes (TCEs), and in more advanced applications (stretchable OPDs), an elastic active layer. Cyclic stressing tests, which include bending, stretching, and compressing treatments, are used to assess the mechanical stability of flexible OPDs. The performance characteristics of recent flexible OPDs with different active materials, flexible substrates, and device performances are summarized in Table 1. In the following section, we will go over the significance and the impact of advances in each of the factors mentioned above.

### 3.1. Flexible Substrate

The first step toward flexible OPDs is to replace rigid glass substrates with thin and flexible ones. Flexible substrates can be made by direct fabrication on plastic foil [55]. Plastic foils such as PET and poly (ethylene naphthalate) (PEN) are frequently used as substrates for flexible OPDs because of their high optical transparency and good barrier properties against gas and moisture. PET and PEN, on the other hand, deform at temperatures above 120–150 °C, making it difficult to deposit indium tin oxide (ITO) electrodes on them without damaging the substrate and this makes the replacement of the ITO electrodes a challenge.

In addition, a polymeric layer spin-coated on a rigid substrate can be peeled off after device fabrication [56]. Using materials with high mechanical strength [57,58,59] or fabricating electronics in a neutral strain position [60] makes it possible to attain very small bending radii (r = ~0.1 mm).

A third very promising route is the use of ultrathin substrates [61,62,63,64]. These substrates offer excellent elastic stretchability and mechanical durability, which are essential for smart skin [65], biological tissue sensing [66], and even solar cells [64]. Polyurethane (PU) [67], poly (styrene–butadiene–styrene) (SBS) [68], poly (styrene-ethylene–butylene–styrene) SEBS [69], Ecoflex [70], polydimethylsiloxane (PDMS) [71] and Parylene C [72] with thicknesses of less than hundreds of micrometers have typically been chosen because of their mechanical elasticity and high transparency.

### 3.2. Transparent Conductive Electrodes

For flexible OPDs to function properly, we need TCEs with low sheet resistance, high optical transmittance, and robust flexibility. ITO and fluorine-doped tin oxide (FTO) are currently used as transparent electrodes because of their high optical transparency (about 90% in the visible region) and electrical conductivity (10–25 Ω sq^−1^). However, ITO is brittle and rigid. A number of materials have been developed to overcome this problem, including carbon nanotubes (CNTs) [73], silver nanowires (Ag NWs) [74], and semiconducting polymers such as poly(3,4-ethylenedioxythiophene) polystyrene sulfonate (PEDOT:PSS (also known as PH1000)) [75].

**Figure 2:**
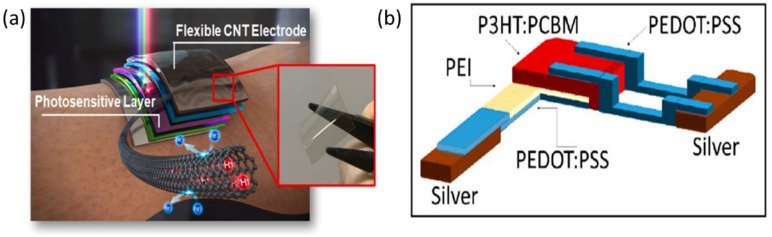
(**a**) Schematic of the device with flexible CNT electrode, reprinted with permission from [23] ©2021 Elsevier.And (**b**) vertical stack of the device with modified PEDOT:PSS flexible electrode, Reprinted with permission from [78] ©2018 ACS Publications.

CNTs have relatively low electrical conductivity (~100 Ω sq^−1^) [76,77]. Recently, CNT TCEs have been optimized by studying the density of CNTs for better optical transmittance and conductivity [23], as shown in Figure 2a. The ideal CNT electrode has a visible transmittance of 90% and a sheet resistance of 98 cm^−2^. Because of the deeper work function, the CNT-based OPDs have a lower J_d_ of 9.62 × 10^−13^ Acm^−2^ compared with ITO or PEDOT: PSS and a higher D* of 2.07 × 10^14^ Jones. The D* after the OPDs were stressed with a cyclic flex test for 500 bending cycles at a bending strain of 0.8 percent only reduced to 80% of its initial value, whereas the D* of ITO-based OPDs reduced to 20% of its initial value.

Solution-processed networks of Ag NWs have a sheet resistance and transmittance comparable to those of ITO (10-25 Ω sq^−1^ at 80% transmittance), together with a relatively high work function of 4.5 eV [79]. Furthermore, they are promising for high-throughput roll-to-roll manufacturing of low-cost transparent conducting films. However, Ag NWs with an electrical conductivity of ≈20 Ω sq^−1^ are required to be processed by nanoimprint lithography [80,81], and their high surface roughness limits their application [82]. Moreover, the Ag NWs that make up the film can easily penetrate the thin active layer (~100 nm) atop the Ag NW electrode, resulting in a short-circuited device. To address this challenge, Yu et al. transferred smooth Ag NW film from a glass substrate to a transparent cross-linked polymer overcoat; Ref. [83] and Gaynor et al. [84] embedded Ag NWs into the conducting polymer PEDOT:PSS by lamination.

PEDOT:PSS is advantageous because of its ease of processing on large areas. It can be deposited as a homogeneous layer by spin- or slot-die-coating or by printing techniques [85]. However, the micro-patterning of this film is not trivial due to the physio-chemical properties of this material and the process’s influence on the TCE’s morphological and electrical properties. In particular, standard photoresists damage PEDOT:PSS films during deposition, development, and chemical removal. In addition, the acidity of PEDOT:PSS also affects the cross-linking or causes uncontrolled de-composition of widely used photoresists. Many different techniques for the photolithographic patterning of PEDOT:PSS have been explored [86]. The most common method is the so-called lift-off process, where a PEDOT:PSS layer is deposited on top of a previously structured photoresist, which is subsequently mechanically removed to give the desired pattern [85]. Another popular approach requires using a sacrificial layer (e.g., parylene or silver) to protect the PEDOT:PSS from the developer. In 2018, Rene Fischer et al. developed a simpler and more effective protocol to structure PEDOT:PSS that does not require any sacrificial layer or specially tailored material [87]. They presented the advantage of intermixing single-wall carbon nanotube (SWCNT) with PEDOT:PSS to form a dispersion for easy deposition. As a result of the low CNT charge, their SWCNT/FE-T (PEDOT:PSS formulation) films present a lower specific resistance (0.26 × 10^−4^ m) than pristine FE-T films (5.6 × 10^−5^ m) while maintaining high optical transparency (91% at 550 nm). The fabricated OPDs eliminate the need for an electron-blocking layer with improved mechanical properties and higher chemical stability than pure FE-T electrodes.

Furthermore, a polyethylenimine (PEI) interlayer was deposited on the PEDOT:PSS layer to modify the cathode’s work function. The use of aliphatic amine-rich polymers, such as PEI, as surface modifiers for electronic contacts was pioneered by Zhou et al. [88]. This helps reduce device dark currents and, therefore, improves the power detection limit and specific detection. In another publication, Matteo Cesarini et al. adopted non-toxic solvents for printing PEI different from the most common 2-methoxyethanol [78], as shown in Figure 2b. Their work studies the PEI interlayer deposition parameter’s heavy impact on the reproducibility of device performance in inkjet printing. Their multi-solvent approach drastically improves yield (from less than 20% to over 90%). In addition, dark currents are reduced to as low as 57 nA cm^−2^. In addition, these devices exhibited a faster response time of up to 30 s, due to a better interface between the PEI interlayer and the photoactive layer.

### 3.3. Active Layers

#### 3.3.1. Organic Materials

Organic semiconductors can be divided into polymers and small molecules. Although both classes of materials achieve similar levels of performance in thin-film transistors (i.e., charge carrier mobility) and solar cells (i.e., efficiency), each material class has a unique set of advantages and disadvantages [89]. For example, polymers can be easier to coat from solution than small molecules [89]. Small molecules, however, are monodisperse and, therefore, are less subject to batch-to-batch variability [89]. The advantage of polymers is that they offer superior mechanical resilience over small molecules since they are van der Waals solids that are not entangled and, therefore, have enhanced strength and toughness. Therefore, the amount of energy absorbed by conjugated polymers during either elastic or plastic deformation, as well as their resilience, tensile strength, or toughness, will likely exceed that of small-molecule semiconductors.

##### Small Molecules

In small molecules, crystallinity has been a key factor in enhancing (opto) electronic performance. In BHJs, crystallinity is linked to enhanced charge photogeneration efficiencies [90]. They typically participate in BHJs as donors, which include diketopyrrolopyrrole (T-DPP-T)-containing molecules, squaraine derivatives, oligothiophenes, acenes, phthalocyanines, various push−pull molecules [90], and molecules with a complicated donor–acceptor (D−A) architecture; see Ref. [91] for a recent review.

**Figure 3:**
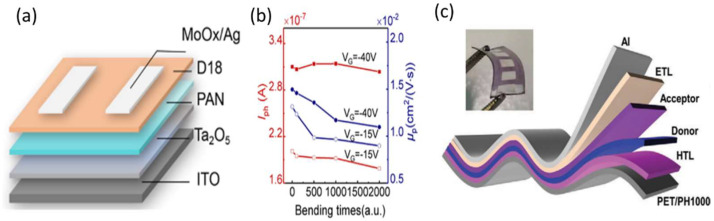
(**a**) Schematic illustration of the D18-based OFETs, (**b**) flexibility test (V_DS_ = −40 V) of the D18-based flexible OPTs, showing how the device mobility and the photocurrent changes under 2000 bending cycles, reprinted with permission from [45] ©2021 ACS Publications. And (**c**) Device structure of flexible SD device, reprinted with permission from [41] ©2021 Wiley.

The higher mobility of small molecules in single crystals makes them better suited for OPTs. On the other hand, OPT development is mainly limited by the following factors: (i) Si and SiO_2_, as commonly used gate electrodes and dielectric layers, respectively, are rigid and fragile. (ii) Channel layers always pose the dilemma of balancing the trade-off between charge transport and light absorption. As a solution to these issues, H. Huang et al. [45] fabricated a phototransistor using high-mobility, unipolar polymer poly[(2,6-(4,8-bis(5-(2-ethylhexyl-3-fluoro)thiophen-2-yl)-benzo [1,2-b:4,5-b’]dithiophene))-alt-5,5′-(5,8-bis(4-(2-butyloctyl)thiophen-2-yl)dithieno[3′,2′:3,4;2″,3″:5,6]benzo[1,2-c][1,2,5]thiadiazole)] (D18). The device architecture was simplified with superior device stability because D18 worked as both the light absorption layer and the charge-transport channel, as shown in Figure 3a. The authors also replaced the SiO_2_ dielectric layers with tantalum pentoxide (Ta_2_O_5_) and polyacrylonitrile (PAN) hybrid layers, which possess high flexibility and capacitance. The calculated photocurrent and mobility values as a function of the bending cycle indicated excellent stability, as shown in Figure 3b.

It is better to construct organic devices with stacked structures for industrial production. The sequential deposition (SD) of donor and acceptor films demonstrates little dependence on the ratio of donor and acceptor materials, solvents, additives, and vertical phase segregation structures, which can simplify device fabrication and achieve comparable power conversion efficiency (PCE) to blend-casting (BC) devices [92]. In 2021, Yanan Wei et al. fabricated a series of self-powered OPDs including D18/2,2′-((2Z,2′Z)-((12,13-bis(2-ethylhexyl)-3,9-diundecyl-12,13-dihydro-[1,2,5]thiadiazolo[3,4-e]thieno[2″,3″:4′,5′]thieno[2′,3′:4,5]pyrrolo[3,2-g]thieno[2′,3′:4,5]thieno[3,2-b]indole-2,10-diyl)bis(methanylylidene))bis(5,6-difluoro-3-oxo-2,3-dihydro-1H-indene-2,1-diylidene))dimalononitrile(Y6) with high D* and photocurrent stability with the SD method [41]. Due to the vertical phase segregation structure, the dark currents (J_d_) were effectively reduced for SD devices, as shown in the schematic in Figure 3c. In a flexible device, the photocurrent and dark currents are 2.2 × 10^−2^ and 3.0 × 10^−7^ A cm^−2^ at 0 V with an R value of 0.35 A W^−1^ and EQE value of 54.43% at 805 nm. In addition, the D* value is over 10^12^ Jones at 665–860 nm with a maximum of 1.14 × 10^12^ Jones at 805 nm. The parameters of the device changed slightly after bending, indicating excellent stability.

##### Polymers

In recent years, the development of conjugated polymers, including BHJs for PDs and flexible phototransistors fabricated with various methods, has been explored rigorously.

In 2020, Canek Fuentes-Hernandez et.al demonstrated a flexible OPD based on a P3HT:ICBA active layer on a polyestersulfone (PES) substrate that achieved a level of performance in the visible spectrum that is statistically equivalent to that of state-of-the-art low-noise Si PDs (Hamamatsu S1133, for example; NEP~200 fW and D*~2 × 10^12^ cm·Hz^1/2^ W^−1^ at B = 1.5 Hz) [93], except for the response time, which is 35 microseconds and remains compatible with video rates. In addition to operating in a large area, the device is capable of producing photodetectors that have complex shapes, such as a ring-shaped flex-OPD, a shape that is better suited to maximizing SNR and minimizing power consumption for PPG sensors. Despite this, the flexible device had lower values of R as a result of series resistance, absorption, and reflection losses (~30%) caused by the semi-transparent Ag/MoOx electrode. In addition, it yielded D* values that were at least two times smaller for flexible OPDs compared to those on glass.

**Figure 4:**
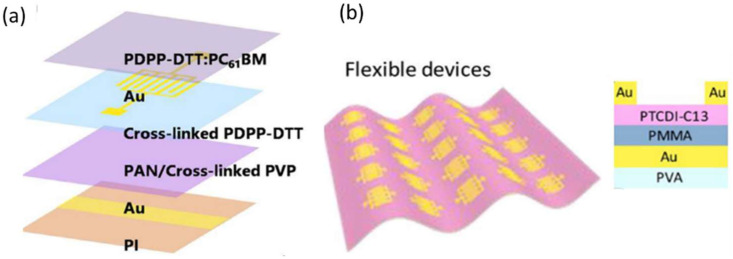
(**a**) Schematics of the flexible OPT reprinted with permission from [44] ©2020 Elsevier. and (**b**) the conformal device reprinted with permission from [104] ©2018 Nature Portfolio.

In BHJs, a large number of additional acceptors easily form scattering centers for hole transport and percolating pathways for electron transport. This results in reduced hole mobility and increased dark current [94]. On the other hand, bilayer heterostructure (BHS) phototransistors have been verified as promising device architectures [94,95,96,97], where the BHS is combined with a BHJ film as the light absorption layer to generate and separate excitons, and a high-mobility semiconductor layer for carrier transport. However, fabricating BHS OPTs in the solution process is challenging because the underlying layer is prone to be destroyed or even washed off during the continuous solution-deposition process. In 2021, Q. Li et al. [44] fabricated NIR polymeric phototransistors with solution-processable BHS, and cross-linked polymeric semiconductors (CLPS) were used as the bottom conducting channel. Additionally, BHJs comprised of low-bandgap polymer donors and fullerene acceptors were used as the NIR photoactive layer; the schematic of the structure is shown in Figure 4a [44]. The active layer, including diketopyrrolopyrrole (DPP)-based polymer (PDPP-DTT), was used to make the phototransistors highly responsive to 808 nm light. They also used indanone-condensed thiadiazolo [3,4-g]quinoxaline-based polymer (PBTTQCN-TT) as the NIR light absorber, which the phototransistors had a good response to, namely 808 nm, 1064 nm, and 1550 nm NIR light. Both devices exhibited improved performance compared with their controlled devices. This strategy enabled the continuous solution processing of multilayer devices and eliminated the limitations of low mobility and high off-current on the performance of phototransistors based on low-bandgap polymers (especially bandgap <1 eV). The flexible NIR phototransistor array was fabricated on a polyimide (PI) substrate. The devices remained relatively stable at different bending radii (as low as 1 mm). Its dark current remained below 0.2 nA even after folding, and its maximum output power (P) was above 10^4^ at a bending radius of 1 mm.

OPTs with conformable channels have utilized p-channel materials due to the lack of n-channel organic materials with air stability and good performance [98,99,100]. The development of conformable OPTs based on n-channel organic material is extremely important for the fabrication of complementary electronics and optoelectronic circuits, which provide various advantages such as high operational stability, easily controlled photo-switching voltages, and high photosensitivity and R [101,102,103]. In 2018, M. Liu et al. [104] presented an ultrathin conformable OPT array on the polyvinyl alcohol (PVA) supporting layer, in which the air-stable n-type N,N′-Ditridecyl-3,4,9,10-perylenedicarboximide (PTCDI-C13H27) thin film serves as the active layer and the thickness of the entire OPT is only~830 nm, as shown in Figure 4b. From the view of molecular design, PTCDI-C_13_H_27_ is a parylene derivative favorable for good stability in n-type organic transistors [10]. The large-area OPT array shows excellent electrical properties in the dark state with mobility as high as 0.58 cm^2^ V^−1^ s^−1^, an extremely high on/off ratio of over 10^9^, and high stability in the air atmosphere. When the OPT surfaces are bent parallel and vertical to channel length, it is found that the performance changes. The on-state current, off-state current, ON/OFF ratio, and mobility are similar, although the conductive channel region presents a different bending deformation. With the decreased bending radius on different curved surfaces, both the dark current and light current of the conformal device present a weak decrease. However, the synchronous decrease in the dark and light current keeps the photosensitivity unchanged and remains at >10^4^ when the device adheres to objects with different curved radii, showing good photosensitivity consistency under strain.

The majority of organic materials absorb visible light and do not have an effective wide-bandgap donor–acceptor material combination. This is why there are no reports of visible–blind UV PM-type OPDs prepared with fully organic materials. To address this challenge, Dechao Guo et.al introduced a novel design for high-performance photomultiplication-type (PM-type) visible–blind flexible UV OPDs based on wide-bandgap organic semiconductor materials (TAPC: C60 blend) [105]. A wide-bandgap absorber was selected as a donor, and a small amount of C60 was used as an acceptor. A device with a ratio of 50:1 displayed a narrowband response, an ultrahigh external quantum efficiency of 1.08 × 10^6^%, and a remarkable specific detectivity of 1.28 × 10^14^ jones at 335 nm under a bias of 14 volts. The flexible device on a PET substrate integrated with a flexible OLED to work as a wearable UV monitor. A key advantage of this configuration is that the flexible visible–blind UV PM-type OPD is capable of driving the OLED without the need for an additional current amplifier since it is capable of generating sufficient photogenerated current under the applied bias voltage and UV illumination. Additionally, the device exhibits UV wavelength-selective response characteristics, so other wavelengths of light will not activate the UV monitor. Furthermore, the wearable UV monitor has excellent weak-light detection capabilities, which are mainly due to the PM characteristics of visible–blind UV OPDs.

##### Dyes

**Figure 5:**
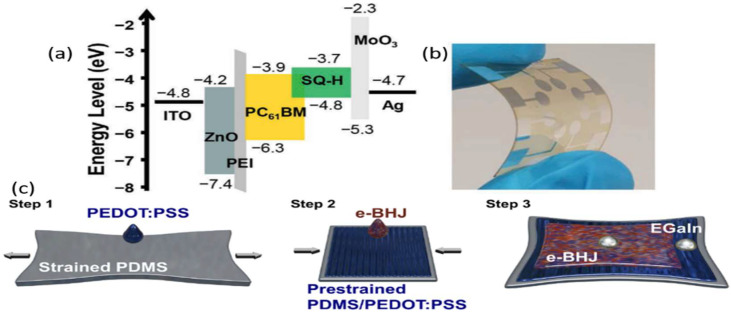
(**a**) Energy diagram of the SQ-H:PC61BM blend-based OPDs, (**b**) photographs of a flexible SQ-H-based OPD, reprinted with permission from [47] ©2021 Wiley, and (**c**) schematics of the fabrication process of the elastomeric OPD, reprinted with permission from [49] ©2021 American Association for the Advancement of Science.

Organic dyes are gaining popularity as alternative photoactive materials due to their tunable optical properties via molecular design and crystal engineering and their inherent high absorption coefficient. Furthermore, their thin active layers are often solution-processable and, thus, flexible and lightweight, and suitable for low-cost wearable applications. J. Kim et al. [47] reported a highly efficient short-wave infrared (SWIR) OPD based on the J-type dicyanovinyl-functionalized squaraine dye (SQ-H) mediated by intermolecular charge transfer; a diagram schematic and the flexible fabricated device are shown in Figure 5a,b [47]. The optimized BHJ OPDs have a maximum EQE value of 12.3% and a full width at half maximum (FWHM) of 85 nm (815 cm^−1^) at 1050 nm under 0 V due to a favorable combination of absorption and charge-transport properties. The flexible devices at both 690 and 1050 nm had a slightly lower performance of 9% EQE.

##### Elastomers

Since the surface of a soft object experiences strains in normal conditions, stretchable PDs might provide an ideal platform for implementing wearable health-monitoring devices such as photoplethysmography (PPG) sensors, [106,107] or sensors mounted onto living organisms for smart agriculture [108]. Furthermore, they can be applied to artificial skin [9,108] and soft robotics [109,110,111], electronic eyes on curvilinear surfaces [112], and asset tracking, gesture, and motion recognition sensors [112]. Elastomeric semiconductors can be used to fabricate stretchable optoelectronics. An elastomeric semiconductor layer has a low Young’s modulus (E) and a large strain at break. Currently, there is no stretchable OPD used in photovoltaics that maintains its performance beyond 50% strain, with one notable exception that sustains 100% strain but has a high E of 5.5 GPa. Recently, a ternary blend of polydimethylsiloxane (PDMS), a donor polymer, and a non-fullerene acceptor yielded a high E value of 990 MPa, maintaining a normalized power conversion efficiency of 86.7% up to a strain of 20% [112]. These devices were tested for photovoltaic applications, so their characteristics were not reported at low irradiance or in the dark. In 2021, Youngrak Park reported combining the elastomer SEBS, the donor polymer poly(3-hexylthiophene-2,5-diyl) (P3HT), and the acceptor Indene-C60 bisadduct (ICBA) to yield a skin-like e-BHJ with a low E and a high strain at break, similar to SEBS [49]. The fabrication process is shown in Figure 5c. Tensile testing on e-BHJ films yields a tensile modulus of 2.4 MPa, with a strain at break of 189%, within the range of values found in human tissue. Further, stretchable OPDs displayed dark current density values smaller than 600 pA cm^−2^ under reverse bias, measured root mean square electronic noise values in the tens of femtoamperes range, and measured NEP values at 653 nm between 13 and 24 pW at strain values up to 60%, resulting in a D* value in the 10^10^ Jones range. The reason was that adding SEBS at 50 weight percent (wt. %) reduces the absorption of light in a pristine BHJ by at least 50%.

#### 3.3.2. Hybrid Active Layers

##### Polymers and Perovskites

Various types of perovskite materials have recently been used for PDs due to their tunable band gaps, long carrier lifetimes, and high light absorption coefficients [113,114,115,116]. Moreover, many studies indicated that flexible perovskite devices could be easily realized on plastic substrates [117,118,119,120] for next-generation optoelectronic devices. However, several challenges must be addressed in this direction, such as developing lead-free perovskite material PDs and finding a better strategy for using metal halide perovskites (MHPs).

**Figure 6:**
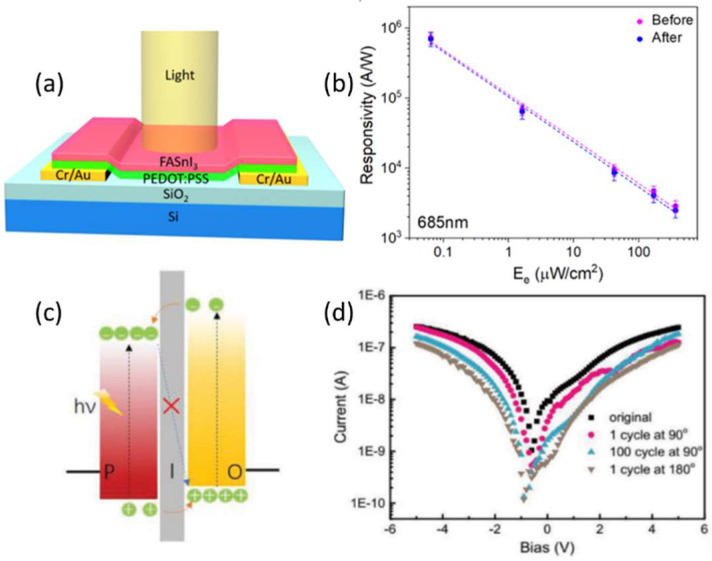
(**a**) Schematic diagram of the FASnI_3_/PEDOT:PSS PD, (**b**) R vs. light intensity for the flexible device before and after the bending test, reprinted with permission from [36] ©2020 ACS Publications, (**c**) schematic illustrating the energy-level diagram of the perovskite insulator organic (PIO) heterojunction and photo-induced charge generation mechanism, and (**d**) the I−V curves of the original flexible PIO device and the device subjected to 1 and 100 bending cycles at 90° and then followed by 1 cycle at 180° under the illumination of white light, reprinted with permission from [48] ©2021 Wiley.

So far, lead-free perovskite PDs have limited R, and their performance is far below the requirement of sensitive detection. Chun-Ki Liu reported a PD based on a lead-free perovskite/organic semiconductor with the structure of [(NH2)2CH] SnI_3_/poly(3,4-ethylenedioxythiophene):poly(styrenesulfonate)(FASnI_3_/PEDOT:PSS) in order to enhance lead-free PD properties with vertical heterostructure as shown in Figure 6a [36]. The device exhibited an excellent photoresponse over a wide range, 300 to 1000 nm, due to a photogate effect. Furthermore, the device’s maximum R and gain are 2.6 × 10^6^ A/W and 4.7 × 10^6^, respectively. Due to the absence of back-scattered light from the substrate, their fabricated device on flexible PI had a slightly lower photoresponse. The device bendability test was conducted 300 times by curving the flexible device to an 8 mm radius on the surface of a bottle. The R at low intensity and the duration of the responses were nearly unchanged, as shown in Figure 6b.

Polycrystalline-built three-dimensional (3D) MHPs suffer from unfavorable charge recombination at grain boundaries and low carrier mobility [121,122,123]. The large numbers of grain boundaries in polycrystalline 3D MHPs act as charge-trapping defects, deteriorating their optoelectronic properties [124]. Moreover, because they are vulnerable to moisture, the grain boundaries are the key factors in the instability of polycrystalline 3D MHPs in the air [124]. As compared to polycrystalline 3D MHPs, 1D single-crystalline MHPs have attracted great interest due to their low light/dark current on/off ratio (>2 × 10^5^), a high R (>400 AW^−1^), defect density, fewer grain boundaries, efficient 1D carrier-transport path, anisotropic 1D geometry, and high flexibility. Despite their promising characteristics, PDs based on pure MHPs have a number of inherent limitations. The most serious problems are the high binding energy and limited carrier mobility [125]. Researchers have considered hybridizing MHPs with other materials to solve these problems to form heterostructures. Such hybridization can improve exciton dissociation, carrier separation, and transportation. Using this approach, various high-performance hybrid MHP PDs have been developed, ranging from perovskite–CNTs [126,127] to perovskite–graphene, perovskite–transition metal dichalcogenides (TMDs) [128], perovskite–Mxene, perovskite–organic heterostructures [120], etc. In 2021, J. Zhang et al. [48] proposed a novel design of radial heterostructure hybrid perovskite microwires (MWs) based on perovskite and an organic semiconductor by a self-assembly process. The charge-transport schematic is shown in Figure 6c. The structure of the radial heterostructure was core MAPbI_3_ MWs fully or partially covered by an assembled P3HT layer with an ultrathin insulating dielectric interface between them. Owing to the efficient exciton dissociation and charge transfer at the huge radial heterojunction interface, the photoresponsivity of the PD based on MAPbI_3_/P3HT hybrid MWs was improved dramatically over that of pristine MAPbI_3_ MWs. The photoresponse of the flexible device was stable under bending conditions, demonstrating the excellent bendability of our perovskite MW-based flexible PDs, as shown in Figure 6d.

##### Polymers and Inorganic Materials

Inorganic semiconductors exhibit higher mobility, a higher optical absorption coefficient, and the ability to be widely tuned by doping, stoichiometry control, size tuning, strain engineering, etc. [52]. However, they require elevated temperatures during device fabrication, require high-cost manufacturing, have limited solution processability, and have less compatibility with lightweight and flexible substrates.

In order to circumvent both organic and inorganic semiconductor limitations, hybrid organic–inorganic junctions are highly desirable. Diverse research groups have used inorganic–organic hybridization for various applications, including UV PDs, piezo-phototronic effects, and IR PDs on flexible substrates.

**Figure 7:**
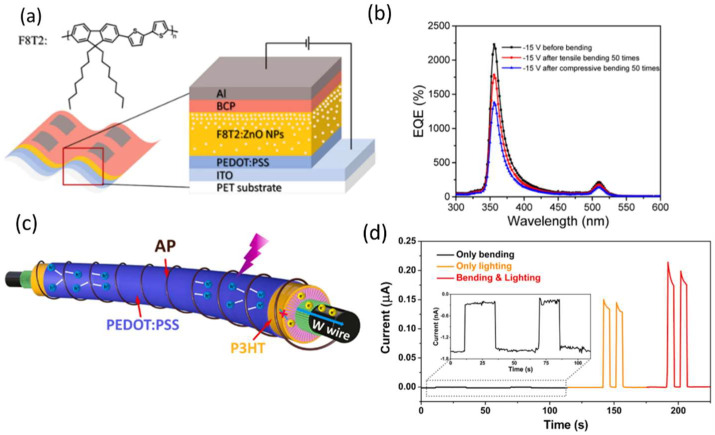
(**a**) Schematic illustration of the flexible narrowband UV PD structure and molecular structure of F8T2, (**b**) EQE spectra of the PD before and after tensile and compressive bending at −15 V bias, reprinted with permission from [129] ©2018 ACS Publications.(**c**) Light-induced carrier transport in fiber-shaped PD based on vertical heterostructure, and (**d**) output current signals of the photodetector under bending, lighting, and lighting + bending, reprinted with permission from [53] ©2021 Elsevier.

In 2018, Xiaoyu Zhang fabricated flexible PM-type narrowband UV PDs using a blend of poly[(9,9-dioctylfluorenyl-2,7-diyl)-alt-co-(bithiophene)] (F8T2) and ZnO nanoparticles (NPs) as the active layer; the structure is shown in Figure 7a [129]. The flexible PDs exhibited EQE spectra with two narrow response peaks under a reverse bias, and a very low dark current density of 1.3 × 10^−5^ mA cm^−2^, even under a strong reverse bias of −15 V. The EQE value produced by the PDs was 2170% under 360 nm illumination and 220% under 510 nm illumination. Moreover, the PDs were bent under both tensile and compressive stresses. The peak D* decreased only by about 5% in total, from 8.8 × 10^11^ to 8.5 × 10^11^ jones, after tensile bending and to 8.3 × 10^11^ jones after compressive bending, as shown in Figure 7b. In both tensile and compressive bending, the response speed remained almost constant.

Fibrous devices are gaining popularity thanks to their light weight, flexibility, wearability, and even stretchability, making them ideal for integrating multifunctional systems such as smart textiles, energy-autonomous electronics, and soft robotics. Fiber-shaped PDs have received little attention despite their low photoresponsivity, slow response speed, and their potential applications. Because of its excellent photoelectric properties, direct bandgap (3.37 eV), and large excitation binding energy (60 meV), 1D ZnO has become the preferred structure for fabricating ultraviolet PDs. Furthermore, the piezoelectric property of 1D ZnO provides a novel strategy for modulating photo-induced charge carrier interfacial transfer. Xinxin Du demonstrated a series of p–n junctions for efficient carrier separation and rapid carrier transport in 2022, using flexible tungsten wire and conductive alginate fiber as the inner and outer electrodes, respectively, with the well-oriented architecture of coaxial multilayers [53]. They reported a self-powered UV PD made from a vertical ZnO/P3HT heterostructure. The structure is shown in Figure 7c. The device was fixed on a flexible PET film to study the bend-induced piezo-phototronic effect. The PD’s output current under lighting and bending (0.19 µA) is significantly higher than that under only lighting (0.14 µA) and only bending (1.35 nA). The bend-induced piezo-phototronic effect due to ZnO’s noncentral symmetric wurtzite structure significantly boosts the photoresponse by increasing the photoresponse by 81.2 percent for the bent device with 1.96 percent strain compared to the device without strain as shown in Figure 7d.

**Figure 8:**
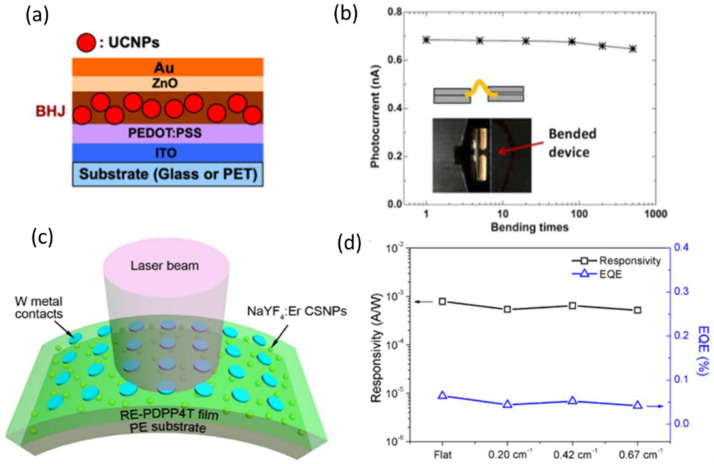
(**a**) Schematic describing the hybrid BHJ (DPPTT-T/PC70BM)/UCNP PD device architecture, (**b**) photocurrent of this device measured after a certain number of bending cycles under the illumination of a λ = 1.5 μm laser at 96 μW reprinted with permission from [140] ©2019 ACS Publications., (**c**) schematic of the planar PD architecture in which the RE−SCP composite rests on a flexible PE substrate, and (**d**) calculated R and EQE at different bending configurations show an unchanged detection response under varying degrees of mechanical stress reprinted with permission from [144] ©2018 ACS Publications.

In recent years, increasing demand for photodetection in the SWIR and NIR spectra for applications such as optical communication, environmental gas sensing, medical diagnostics, night vision, light detection and ranging (LIDAR) [130], and long-term wearable monitoring has been utilized. However, many IR-responsive materials are rigid and unstable in air, which limits their use. Conjugated semiconducting polymers have aroused much interest for their application in low-cost photodetection [130]. While there is continuous progress toward synthesizing low-bandgap polymers, it is still difficult to have SWIR photodetectors entirely based on semiconducting polymers exhibiting R beyond λ = 1.2 μm [131,132,133,134]. One possible strategy to reach that point is to apply photon upconversion to convert low-energy photons into high-energy ones. Various upconversion systems in the form of solution-processed NPs have been synthesized, [135,136,137] mostly based on the doping of trivalent lanthanide cations in low-phonon-energy hosts, which perform upconversion through a two- or multi-photon mechanism [138]. Recently, Zhang et al. fabricated novel erbium silicate nanosheets to upconvert λ = 1.5 μm photons to visible ones [139]. In 2019, Hengyang Xiang et al. demonstrated high-performance flexible photodetectors sensitive to λ = 1.5 μm photons based on the formation of a solution-processed organic (a donor–acceptor BHJ blend of diketopyrrolopyrrole (DPP)-based copolymer and [6,6]-phenyl-C71-butyric acid methyl ester (PC71BM))/inorganic hybrid composed of conjugated polymer/small-molecule bulk heterojunctions (BHJs, host) together with Er3-doped upconversion nanoparticles (UCNPs, guest); its structure is shown in Figure 8a [140]. Under the illumination of λ = 1.5 μm SWIR photons, optimized hybrid BHJ/UCNP photodetectors exhibit a clear photoresponsivity of 0.73 and 0.44 mA/W for devices built on glass substrates and flexible PET substrates, respectively. The rise time of the device was 80 μs under the illumination of λ ≈ 1.5 μm photons, which is faster by more than one order of magnitude than previous photodetector studies applying Er3+-doped upconversion systems [139] and by two to four orders than most other non-avalanche semiconductor photodetectors at SWIR wavelengths [141,142,143]. Mechanical bending tests showed a bending angle greater than 120°, and a photocurrent with minimal photocurrent change (≈5%) after 500 bending cycles, as shown in Figure 8b. In another publication, Xinyu Zhao et al. reported, for the first time, a flexible SWIR photodetector with excellent photosensitivity to eye-safe light at 1532 nm using a SWIR-responsive rare earth doped-nanoparticle semiconducting polymer (RENP−SCP)(RE−SCP) composite; its schematic shown in Figure 8c [144]. The RE−SCP composite maximizes photon-to-electron conversion efficiency and minimizes optical scattering losses through (1) spectral matching of the properties of SWIR-responsive nanophotonic converters with that of an SCP and (2) sophisticated control of nanophotonic converter size and interactions within the SCP matrix. The SWIR upconversion characteristics of the NaYF_4_ core−shell NPs (N-NPs) that dictate the success of SWIR detection of these RE−SCPs were controlled by tuning the dopant chemistries to match the SCP’s peak absorption properties. Subsequently, an optimal photoactive layer of SWIR-responsive N-NPs dispersed within a diketopyrrolopyrrole-based SCP was fabricated. The decrease in photocurrent density J was observed for the photodetectors with different bending curvatures because of the reduction in incident illumination power density due to an increase in the illumination area for the bent photodetector. The calculated R and EQE, with minimal differences for different curvatures, are shown in Figure 8d.

The development of flexible and long-term stable NIR phototransistors using low-cost processes has remained a challenge. Dingwei Li fabricated hybrid phototransistors using n-type metal oxide/polymer semiconductor heterostructures: indium oxide (In_2_O_3_) and n-type polymerizing small molecular acceptors (PSMA), a polymer semiconductor of poly5,5′-bis[3,5-bis(thienyl)phenyl]-2,2′-bithiophene-3 ethylesterthiophene] (PTPBT-ET) [46]. By virtue of rational energy band alignment, the hybrid phototransistor combined the advantages of both In_2_O_3_ transistors and the high NIR-response of PTPBT-ET polymers, exhibiting a high saturation mobility of 7.1 cm^2^ V^−1^ s^−1^ and a large current on/off ratio of >10^7^. The phototransistor performed well in NIR light sensing, with an R of 200 A W^−1^, a D* of 1.2 × 10^13^ Jones, and a fast photoresponse time of 5/120 ms. Moreover, even after 160 days in the air, their device maintained excellent electrical stability with no performance degradation. The device bending tests showed that with a bending radius of 5 mm, device characteristics remained unchanged for up to 1000 bending/releasing cycles.

##### Polymer and Two-Dimensional-Based Materials

Two-dimensional materials and their three-dimensional derivatives show extraordinary optoelectronic and photodetection properties [145,146,147]. On the other hand, due to their stiffness and instability under air exposure, they need to hybridize with other soft semiconductor material systems to be applicable in flexible applications.

Among these two-dimensional semiconductors are layered MoS_2_, WS_2_, MoSe_2_, InSe, black phosphorous, SnS_2_, SnSe_2_, and GaSe, which are being investigated extensively for optoelectronics as they exhibit high absorption coefficients and tunable properties. Among these layered materials, SnSe_2_ is a potential candidate for optoelectronic applications owing to its high intrinsic absorption coefficient [148] and being an earth-abundant and environmentally benign compound. SnSe_2_ exhibits a hexagonal CdI_2_ crystal structure with n-type conductivity and a direct bandgap of around 1.2 eV. In 2022, B. Reddy et al. [52] fabricated a bulk heterojunction (BHJ) of SnSe_2_–PEDOT:PSS by simple sonication-assisted mechanical mixing for broadband photodetection. The flexibility tests showed that the measured R values exhibit minimal degradation in performance due to bending. The response time was estimated to be 16 ms, 221 ms, and 274 ms for UV, Vis, and NIR spectra, respectively, and it was higher for the UV light due to the band transition in ITO, which has a bandgap of 3.38 eV. Furthermore, the device showed detectivity values of 6.51 × 10^10^ Jones, 6.23 × 10^10^ Jones, and 6.08 × 10^10^ Jones in the UV, visible, and NIR regions, respectively. Moreover, the device exhibited good stability after 4 weeks and retained ~90% of its initial performance.

**Figure 9:**
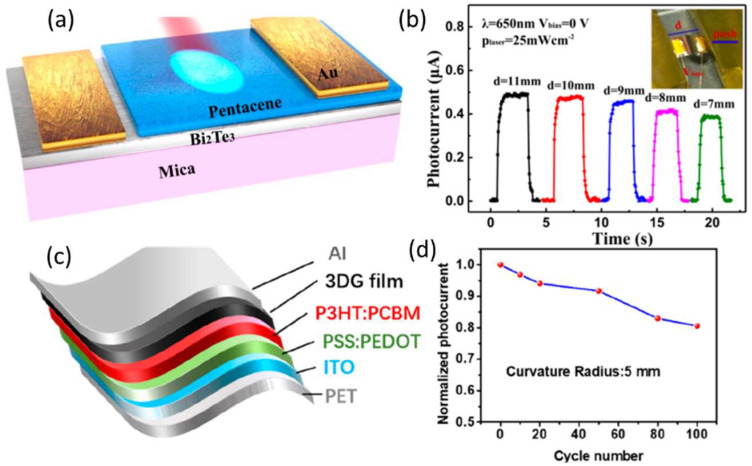
(**a**) The structure diagram of Bi_2_Te_3_/pentacene heterostructure self-powered PD, (**b**) the distance between two ends and photocurrent diagram of PDs under irradiation of 650 nm, reprinted with permission from [154] ©2019 ACS Publications. (**c**) schematic of the flexible 3DG film/OPD, and (**d**) normalized photocurrent for flexible 3DG film/OPD as a function of cycle number of repeated bending to a radius of 5 mm reprinted with permission from [50] ©2022 ACS Publications.

The most appropriate three-dimensional topological insulators (3D TIs), Bi_2_Te_3_, with a suitable narrow bandgap (0.145 eV), have drawn considerable attention [149,150,151,152,153]. Although some progress has been made, photodetection based on Bi_2_Te_3_ is usually hindered by the large dark current and dilatory response time due to the lack of inherent differences. Moreover, the above Bi_2_Te_3_-based photodetectors are in the IR region, specifically in the mid-IR range. In 2019, Ming Yang et al. prepared a photodetector with a 3D TI Bi_2_Te_3_/organic heterostructure using n-type Bi_2_Te_3_ and p-type organic thin films, and the structure is shown in Figure 9a [154]. This photodetector possesses a wide-waveband response from VL to mid-IR (450–3500 nm) with an ultrahigh R up to 14.89 AW^−1^ and an ultrafast response time of 1.89 ms at room temperature. More importantly, the R reaches 1.55 AW^−1^ at 3500 nm, several orders of magnitude higher than previous photodetectors based on 3D TIs. At the same time, the response time is one order of magnitude faster than that of the previous Bi_2_Te_3_-based photoelectric devices. Devices constructed on a flexible mica substrate exhibited a photocurrent diagram at 650 nm in the self-powered mode when a bending force was applied at two sides of the device. As the force increases, the magnitude of the photocurrent decreases as the distance between the two sides d decreases. When the device is at its maximum bending (4 mm), the photocurrent decreases by only 9.8%, indicating the flexibility and stability of the device, as shown in Figure 9b.

To achieve high-responsivity NIR and mid-infrared (M-IR) detection, it is critical to introduce appropriate materials with efficient NIR and M-IR light absorption. Single-layer graphene exhibits potential NIR and M-IR detection at room temperature, making it appealing for broadband and flexible photodetectors. Nonetheless, practical application is limited by graphene’s low light absorption, high noise, and high dark current, as well as the stability and toxicity of QDs, the high gating voltage, and the complex phototransistor structure. Three-dimensional graphene (3DG) sponge, which is composed almost entirely of edge-linked single-layer graphene sheets and behaves in a similar way to a true bulk graphene material, has a high specific surface area and broadband absorption characteristics, making it an excellent light detection material. On the other hand, photoconductors have low R since photogenerated electron–hole pairs cannot be effectively separated. Furthermore, thin 3DG has poor mechanical properties and a porous surface, making it difficult to handle and maintain good electrode contact. In 2022, Zhen Ge reported a flexible Vis–M-IR photodetector in OPDs using a hybrid layer of 3DG film and classic PCBM- and P3HT-based BHJ materials; the structure is shown in Figure 9c [50]. Flexible 3DG film/organic hybrid detectors are compatible with flexible substrates, and the dark current of the flexible device is slightly lower than that of the rigid device due to the higher resistivity of PET/ITO. The response current of the flexible device can maintain more than 90% of that of the rigid device under 520 nm illumination. Furthermore, after 100 bending cycles, the flexible device’s photocurrent still maintains more than 80% of the initial response current, as shown in Figure 9d.

## 4. Applications

**Figure 10:**
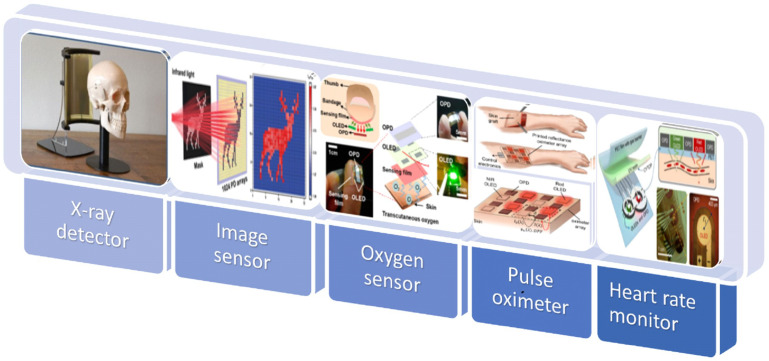
Schematic illustration of representative applications of organic material flexible photodetectors.

This section discusses the application of OPDs to wearable electronic devices, such as health-monitoring devices, image sensors, and X-ray detectors. In Figure 10, a pictorial description of the flexible OPDs is shown.

### 4.1. Health Monitoring

Heart rate monitors, pulse oximetry sensors, and transcutaneous oxygen (oxygen level of tissues beneath the skin [155]) monitors all use PDs. OPDs have been used in noninvasive and low-cost PPG sensor technology, for example. The PPG sensor detects changes in blood flow volume in a microvascular bed of tissue, such as in the fingers. The PPG signals are measured by using OPDs to track changes in light intensity. PPG sensors can be designed in two modes: reflection (aligning the light source and OPD side by side) or transmission (placing the light source and OPD on two sides of a finger). For the optical implementation of heart rate sensing, a highly detective photodetector is required because human tissue (bones, blood, fat, water, and melanin) scatters and absorbs a large portion of light. In 2018, G. Ryu et al. [156] reported a printed flexible optoelectronic sensor composed of red organic LEDs (OLEDs) and organic PDs (OPDs) for fabricating flexible PPG devices and the subsequent detection of various biological signals. Three OPD structures were investigated. PPG signals were successfully detected from the fabricated flexible PPG sensor and driving circuit. A human study was conducted to evaluate the flexible PPG sensor’s performance in a real-world application, where subject drowsiness was estimated from the PPG signals. Heart rate variability (HRV) was extracted from the PPG signals using machine learning algorithms to classify drowsiness. The flexible PPG sensor produced 79.2% accuracy and 72.1% area under the receiver (AUC) to predict drowsiness, which are meaningful results compared to conventional PPG sensors (83.3% accuracy and 69% AUC).

**Figure 11:**
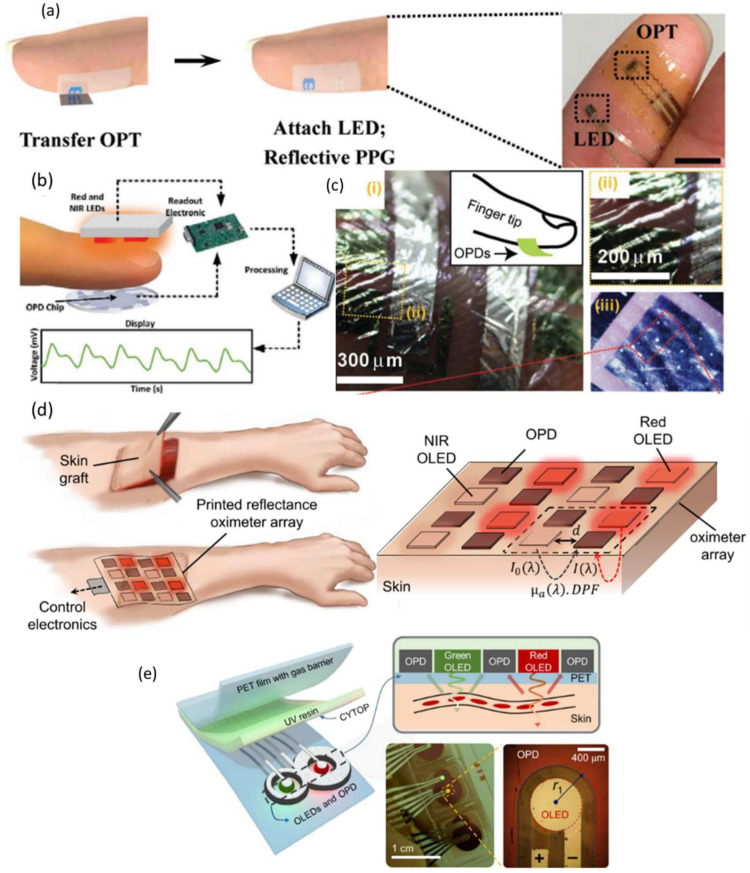
(**a**) Photograph of a finger covered with the epidermal hPPG sensor (scale bar, 5 mm). (**b**) Illustration of the testing setup used for PPG waveform acquisition, (**c**) skin conformal near-IR photoplethysmogram sensor. (**c**) (i, left image) Photograph of fingerprint-conformal near-IR PDs; inset indicates position of skin-conformal near-IR PD on finger, (ii, right top image) enlarged image of (i), and (iii, right bottom image) microscope image of top silver electrode and skin surface, indicating high skin conformability, Reprinted with permission from [158] ©2017 Wiley. (**d**) Schematic of an application scenario of the ROA: 2D oxygenation mapping of a skin graft on the forearm. After surgery, the ROA is placed on the skin graft to map oxygenation of the reconstructed skin and ROA sensor configuration, reprinted with permission from [164] ©2018 American Association for the Advancement of Science. (**e**) Schematic of the proposed OPO sensor with enlarged cross-sectional view to depict device arrangement and light-receiving process through the skin medium, reprinted with permission from [163] ©2018 National Academy of Sciences.

The all-organic devices provide great flexibility and conformity to the skin; however, they currently only operate in the visible spectral range (e.g., green and red). This imposes a limitation on the penetration depth of light into the skin tissue and limits the ability to detect signals. In addition to penetration depth, light attenuation also needs to be taken into account. To engineer an OPD with high EQE in the red-NIR spectrum, the photosensitive organic semiconductors (OSCs) must have a low optical bandgap, ideally ≈1.20 eV or lower [157]. In 2017, H. Xu et al. [158] demonstrated epidermal and flexible PPG sensors that could operate in the NIR regime with high sensitivity and low power consumption. The configuration of the PPG sensor is shown in Figure 11a. The PPG sensor hybridizes an organic phototransistor (OPT) with an inorganic light-emitting diode. This LED offers much higher power conversion efficiency (14.3%) compared to NIR OLEDs, and it can be easily bonded to flexible substrates through transfer printing.

In 2022, João Simões et al. [51] demonstrated a new solution-processed flexible OPD architecture based on a BHJ of poly[4,8-bis(5-(2-ethylhexyl)thiophen-2-yl)benzo[1,2-b;4,5-b′]dithiophene-2,6-diyl-alt-(4-(2-ethylhexyl)-3-fluorothieno[3,4-b]thiophene-)-2-carboxylate-2-6-diyl)] (PTB7-Th) and 2,2′-((2Z,2′Z)-(((4,4-bis(2-ethylhexyl)-4H-cyclopenta[2,1-b:3,4-b′]dithiophene-2,6-diyl)bis(4-(heptan-3-yloxy)thiophene-5,2-diyl))bis(methanylylidene))bis(5,6-difluoro-3-oxo-2,3-dihydro-1H-indene-2,1-diylidene))dimalononitrile (COTIC-4F). The narrow optical bandgap of the NFA COTIC-4F allowed good photoresponse beyond 800 nm, outperforming the majority of fullerene-based OPDs. Blood oxygen saturation was monitored successfully using a custom PPG sensor integrating the fabricated OPD, which achieved a low error of 2% compared to a commercial PPG sensor. The configuration is shown in Figure 11b. The resultant PPG waveforms, between 40 and 50s for 660 and 940 nm, showed from ≈21 to ≈22 mV with a ≈1 mV_p-p_ peak to peak, and from ≈16.5 to 18 mV with a ≈1.5 mV_p-p_ peak to peak, respectively. They mentioned that since the HbO_2_ absorbs less red light than NIR light, in cases where SpO_2_ is high, blood volume changes have less influence on the detected red signal.

In 2018, Sungjun Park et al. reported skin-conformal NIR OPDs that simultaneously achieve a high cutoff frequency over 1 kHz at −3 dB and mechanical conformability sufficient for health-monitoring applications [107]. The configuration is shown in Figure 11c. The ultrathin (total thickness < 3 µm) organic photodetector showed exceptional operational durability under conditions of extreme mechanical deformation (10^3^ cycles of operation at a bending radius of 3 µm), with stable charge carrier transport under repetitive motion. The precise and fast near-IR switching behavior with low dark current could be controlled by modulation of resistance and capacitance of the active layer. In addition, the long-term near-IR responsive operation is maintained even when the device is attached to a curved and/or folded part of the human skin by locating devices at a neutral plane within 3 µm of device’s total thickness.

Noninvasive 2D oxygenation mapping capability has the potential to transform real-time and post-surgery management as well as the monitoring of wounds, tissues, and organs [159,160,161,162,163]. An in vivo spatial oxygenation mapping device can aid in assessing tissue damage and injury susceptibility. One such application scenario, where a flexible optoelectronic sensor array is used to map the 2D oxygenation of a skin graft, is illustrated in Figure 11d. In 2018, Y. Khan et al. [164] reported a reflectance oximeter array (ROA): a flexible and printed electronic system realized by printing and integrating arrays of organic optoelectronics with conventional silicon integrated circuits for blood and tissue oximetry. The ROA is composed of four red and four NIR printed organic light-emitting diodes (OLEDs) and eight organic photodiodes (OPDs). The fabricated sensor on flexible plastic substrates is comfortable to wear and increases the SNR by establishing a high-fidelity sensor–skin interface. With a 0.7 × 0.7 cm^2^ active area for both OLEDs and OPDs and 0.5 cm spacing between the OLEDs and OPDs, the dimensions of the complete ROA became 4.3 cm in both length and width. The cutoff frequency was measured at over 5 kHz for OPDs. Since the operation frequency of the pulse oximeters is generally less than 1 kHz, this bandwidth is sufficient for oximetry. Additionally, they explored the placement of the sensor on the body. The forehead provided the strongest pulsatile signal, and their reflectance oximeter monitored the oxygen saturation of a volunteer on the forehead with a mean error of 1.1%.

Most organic-based pulse oximeters focus more on the feasibility and/or on such form factor advantages. Little attention, on the other hand, has been paid to the potential of organic-based pulse oximeters in terms of ultra-efficient operation to meet the challenging power demand of wearables applications. In 2018, Hyeonwoo Lee et al. showed a reflective patch-type OPO sensor with ultralow power consumption based on flexible OLEDs and OPDs [165]. The configuration is shown in Figure 11e. Through an optical simulation, an ideal OPD wraparound layout has been proposed where an OPD resembling the numeral 8 wraps around small circular OLEDs for each red and green emission. With this approach, the proposed monolithically integrated OPO sensors exhibited successful operation at electrical powers as low as a few tens of microwatts on top of various body parts. Moreover, the conformal, index-matched contact between the substrate and the skin makes it possible to access and use a significant amount of light that would otherwise be confined within the substrate. It was also shown that this index matching is important for the suppression of direct coupling of light between an OLED and an OPD. The results presented here illustrate that organic devices not only have form factor advantages but also hold great promise as enablers for all-day wearable health-monitoring systems.

Opportunities in the seamless, continuous assessment of health/wellness and advanced functions in wound monitoring/care and human–machine control systems motivate research in this field. Babilas et al. [166] measured transcutaneous O_2_ pressure (tcpO_2_) under variations in the microcirculatory system using a luminescent O_2_ sensor [166]. However, the tcpO_2_ value was recorded using a luminescence lifetime imaging method, which requires expensive devices apart from the sensing film on the skin [166]. Additionally, Liu et al. [166] demonstrated a structurally integrated luminescent O_2_ sensor composed of OLEDs, an OPD, and sensing film [166]. However, the proof of concept was limited to the detection of dissolved [O_2_] [166]. In 2018, Chang-Jin Lim et al. presented a new concept for a wearable tcpO_2_ sensor utilized in the early detection of diseases based on O_2_ partial pressure by combining the technologies of luminescent gas-sensing, wearable, and biocompatible devices. The sensor was a bandage-like O_2_ sensor based on the luminescent sensing mechanism and consisted of three components on a plane: a sensing film, an OLED, and an OPD as a detector. As the sensor facilitates the time-resolved, real-time monitoring of patients, the number of medical staff members required to care for a patient can be drastically reduced, facilitating the immediate care and rescue of patients. In addition, there is little need for patients to be hospitalized for this real-time monitoring, which will result in significantly reduced medical costs. The I_0_/I_30_ (2.19) of the sensor was sufficient for measuring tcpO_2_ variations in tissues of the lower arm and a thumb after pressure-induced occlusion, and due to its noninvasive and flexible features, it could be used in any part of the body, even when a person is exercising or working.

### 4.2. Image Sensors

One advantage of OPDs in comparison with conventional inorganic devices for imaging is the ease of achieving full-color detection. For conventional photodetectors, which have a broad absorption range covering the entire visible spectrum, color selectivity is typically achieved using red, green, and blue filters. Filters, however, reduce the amount of light, limiting detectability [167].

**Figure 12:**
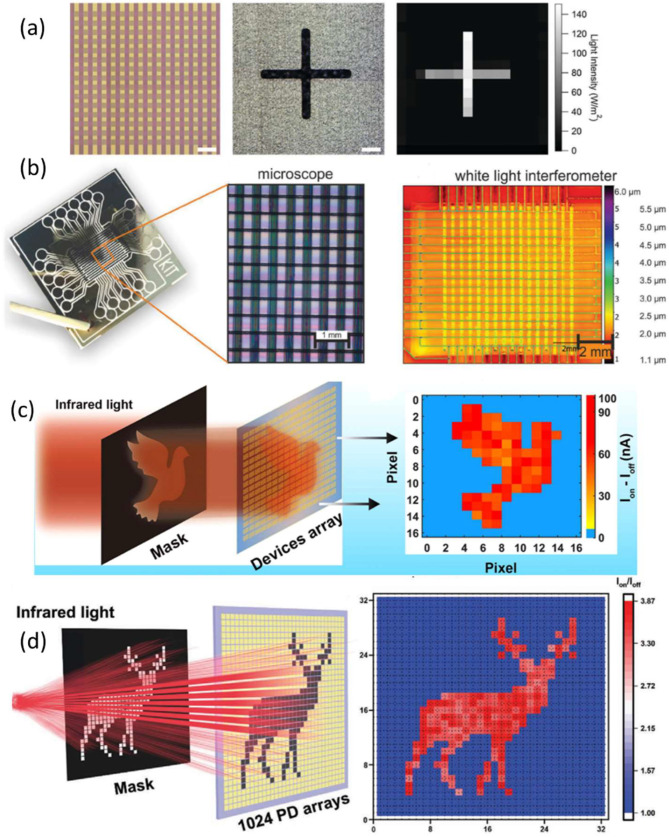
(**a**) Optical microscope image of a 16 × 16 (active area, 2.40 mm^2^; pixel pitch, 50 µm, left panel) array on a glass substrate, Reprinted with permission from [168] ©2018 Wiley, (**b**) photograph, microscope image, and topographical image (white light interferometric microscope image) of the completed pixel array, reprinted with permission from [171] ©2018 Wiley, (**c**) schematic illustration of the image sensor irradiated by 1342 nm light and the corresponding output images of the bird by the flexible SWIR image sensor, reprinted with permission from [39] ©2020 Elsevier, and (**d**) schematic diagram of multi-pixel image sensor imaging and the deer pattern composed of 1024-pixel output by Ti_3_C_2_Tx-RAN PD image sensor reprinted with permission from [54] ©2022 Wiley.

Increased pixel densities are an important feature of sensing arrays, and to date, minimizing pixel footprints has been challenging. The ratio of the sensor’s active area to the total pixel area is called the pixel fill factor [112]. For image sensors, fill factors of less than unity result in poorer sensitivity and increased cost, requiring the use of micro-lenses to direct light to the sensor [168]. Thus, monolithic integration is preferred since it would allow for pixel fill factors of unity—pushing achievable pixel densities to their limit. The major challenge in manufacturing these types of monolithic architectures is maintaining the process compatibility of all layers during the fabrication process. In 2018, Peter Zalar et al. demonstrated a passively addressed photodiode matrix that takes advantage of the monolithic integration of photodiodes and diodes [169]. Their structure permits high pixel densities shown at up to 262 pixels per inch (ppi), with pixel areas of just 2.5 × 10^−3^ mm^2^ and pixel pitches of 50 µm. Imaging capability is shown in Figure 12a. This is possible because a pixel fill factor of unity can be maintained by virtue of this structure. The demonstrated structure allows for real-time imaging with a satisfactory resolution for a variety of applications such as real-time spatial PPG measurements on the ball of a human foot and heart rate measurements matching simultaneous electrocardiography (ECG) measurements.

Due to limited registration accuracy, variation in the printing process stability, and highly sensitive drying effects [170,171], the imager device integration and packing density, in combination with consistent device performance, have remained challenging. In 2018, Ralph Eckstein et al. overcame these challenges by exploiting a self-alignment process induced to fabricate a fully digitally printed image sensor based on organic photoactive materials with high performance, reproducibility, and lab-scale fabrication yields of 100% [172]. The passive matrix image detector is composed of 256 micro-pixels with individual active areas of ~250 µm × 300 µm for a total footprint of 64 mm^2^; the imaging capability is shown in Figure 12b. Characterization of the single OPD pixels demonstrated state-of-the-art performances. The robust fabrication method was based on printed dewetting patterns, which improves registration accuracy and reduces film-forming defects.

Lei Lv used their single-polymer PD to create IR imaging sensors in another study [39]. The polymer PDs were incorporated into a 256-pixel (16 × 16) flexible image sensor with the number of pixels easily increased to a million-scale array using an industrial micromachining technique. One pixel has a sensor area of 900 × 900 µm and a periodicity of 700 µm. With irradiation of 1342 nm (26.3 mW/cm^2^) at 8 V, the corresponding patterns were placed on the array’s surface to display the expected images. Imaging capability is shown in Figure 12c.

In some works, researchers tried to use hybrid heterostructures to enhance the performance of the imagers. As an example, Chuqiao Hu reported a high-pixel imaging system with 1024 pixels (32 × 32) selected from the Ti_3_C_2_Tx-RAN PD array and integrated into the flexible image sensor; the imaging capability is shown in Figure 12d [54]. The incident light (1064 nm, 159 mW cm^−2^) integrated into the flexible image was irradiated from the backside of the substrate during the image-sensing measurement, and the deer-shaped mask was placed on one side of the device array. The pixels were all responsive to the 1064 nm light, except for the area covered by the mask. The image is clearer, and the deer pattern is more vivid and exquisite, thanks to the thousands of pixels. The high imaging capability of Ti_3_C_2_Tx-RAN PDs suggests that they have a lot of potential in the field of high-pixel image sensing.

### 4.3. X-ray Detectors

**Figure 13:**
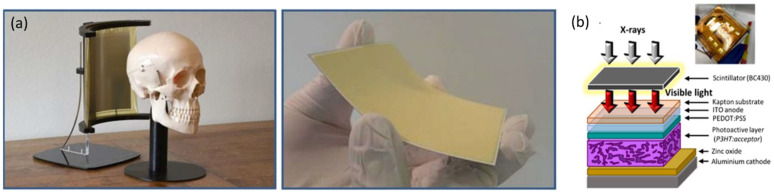
(**a**) Prototype of curved image sensor, Reprinted with permission from [38] ©2020 Nature Portfolio and (**b**) layered device architecture employed in the fabricated organic photodetector devices of this study. The scintillator is employed to facilitate indirect X-ray detection for dosimetry characterizations Reprinted with permission from [42] ©2021 ACS Publications.

Medical imaging, nondestructive testing in industrial products, and public security inspection are just a few examples of where X-ray imaging is used. Indirect conversion X-ray detectors that combine scintillator and visible OPDs are used to detect X-rays using OPDs. The X-ray excitation activates the scintillators, which then emit visible light. OPDs have been designed to match the luminescence of scintillators in terms of spectral response. However, the absorption of light by the organic semiconductor forms excitons, which need to be dissociated, resulting in significant losses and limiting detector sensitivity as opposed to a direct conversion process. In 2018, H.M. Thirimanne et al. introduced a broadband, direct, X-ray detector concept based on a thin-film hybrid semiconductor diode consisting of an organic BHJ–bismuth oxide (Bi_2_O_3_) NP composite [173]. The flexible detector offers a high sensitivity of 280 µC mGy^−1^ cm^−3^. More importantly, these sensitivities are achieved at −10 V. The improved X-ray sensitivity is a result of impact ionization and an enhanced path length due to Mie scattering and the efficient separation and transport of these by the BHJ–NP architecture, resulting in high charge collection efficiencies (>60%). Because of its rigidity, the traditional X-ray detector is limited in its application in curved sensors. Two-dimensional spatial detection and self-powered operation are enabled by a flexible X-ray detector made by combining the printed OPD with a plastic scintillator. A plastic foil-based curved digital X-ray image sensor developed for approaching cone-beam computed tomography X-ray imaging is shown in Figure 13a [38]. The curved X-ray image sensor has a large surface area of 6.0–8.0 cm and 480 × 640 pixels. The X-ray image sensor successfully captured a full 3D volume image of a piece of bone. Over the entire area of the digital detector, the images show good and homogeneous contrast. Because the lateral spread of optical photons is lower in the thinner scintillator layer than in the thicker scintillator, the spatial resolution is clearly higher. For the 700 and 400 µm thick scintillators, the modulation transfer function (MTF) was 31 and 37 percent at 1 lp/mm, respectively. These figures match the MTF predicted for an X-ray detector with a 40 µm thin-film barrier between the image sensor and the scintillator.

Fabricated flexible OPDs have been reported to show fluorescence under the high-intensity X-ray radiation utilized in medical applications [174]. This radiation-induced fluorescence produces a strong non-linearity in the R of the OPDs due to their high sensitivity to optical photons and prevents the full realization of tissue-equivalent printed polymer dosimeters. M.J. Large et al., in 2021, provided the first study of a non-fullerene acceptor (o-IDTBR) in combination with a donor polymer fabricated onto polyimide (Kapton) as a mechanically flexible substrate that is known to exhibit high transmission to X-rays and negligible radiation-induced fluorescence; the structure is shown in Figure 13b [42]. To enhance the sensitivity of the organic detectors, they utilized a plastic scintillator with a spectral window where the organic semiconductors have a much higher absorption coefficient. Full characterization of the flexible OPD device response to both visible light and X-rays indicated acceptable tissue equivalence, good sensitivity to X-rays, and also demonstrated a large improvement in stability under high radiation doses when utilizing the non-fullerene-based o-IDTBR in place of the more widely studied PCBM. This sample preservation method resulted in negligible variations in the photoresponse of the detectors over a time period of 17 days. Detectors employing photoactive layer P3HT:o-IDTBR displayed an extremely linear response with increasing radiation dose with indirect X-ray detection sensitivities 114.2 ± 0.7 pC/cGy. In another publication, in 2021, Jessie A. Posar et al. reported the first-ever demonstration of a printed X-ray detector that is fully tissue-equivalent, has a rapid temporal response, and exhibits good sensitivity at zero-bias operation [175]. This performance is achieved by coupling an RP400 plastic scintillator with a photodiode composed of donor polymer P3HT and NFA (o-IDTBR) to create an indirect X-ray detector. The X-ray-detecting material system was printed into flexible devices with pixel sizes of 60 μm that exhibit exceptional stability against degradation due to aging, repeated bending, and high-irradiation doses. PET films are known to exhibit scintillation with a deep-blue photon emission, potentially overlapping with that of the RP400 plastic scintillator employed in the devices [176]; however, based on their investigation, these barrier films have minimal effects on both sensitivity and radiation hardiness [176]. The device showed no degradation after encapsulation for up to 1000 h within the measurement error margin of 4%.

## 5. Discussion

Flexible OPDs based on solution-processable technologies provide a significant cost benefit and functional advantage over wafer-based PD techniques by surpassing their natural issues such as strong exciton binding and non-crystalline structures. These advantages are obtained from the chemical composition of the photoactive layer (novel organic materials or hybridization) or from modifying the device structure.

A flexible OPD’s bending radius and D* are both key figures of merit which can be considered for classification when evaluating their applicability. Among the studied flexible OPDs, the one with D18:Y6 BHJ and on PET substrate [41] shows the highest bending radius of 11 mm due to the more flexible nature of PET in comparison to other flexible substrates. However, it exhibited a moderate D* of 1 × 10^11^ Jones. In terms of D*, both advanced organic active materials and their hybrid counterparts perform very well by reaching values more than ~1 × 10^13^ Jones [43,50,51]. Moreover, utilizing novel functional layers such as new electrodes and interlayers has resulted in the highest detectivity of 1 × 10^14^ Jones for flexible organic photodiodes based on CNT electrodes [23]. In another class of flexible OPDs, conformal devices show promising stretchability by tolerating ~60% of applied strain and losing minimal performance after bending [49]. Nonetheless, their D* still needs to improve for further feasibility since they show a relatively low value of ~1 × 10^10^ Jones due to their elastomeric active materials with lower absorption coefficients.

On the other hand, several critical challenges regarding the real-world commercialization of flexible OPDs remained unsolved. First, although flexible OPDs with good resistance to water [159], heat [177], and mechanical deformation [78] have now been demonstrated, even better output stability and device lifetime are likely preferred. Thus, further development of scalable and low-cost encapsulation strategies and ultrathin passivation layers that do not compromise the mechanical flexibility of the device are needed. Second, the integration of flexible OPDs with other electronic components (such as batteries, wireless transmitters, and read-out integrated circuits) is essential for fully integrated electronic systems. Ultimately, developing OPDs that can be integrated into textiles and polymer fibers would be a promising route for application in health monitoring and telecommunications.

## 6. Conclusions

The recent progress toward the realization of high-performance and mechanically flexible OPDs has been highlighted. The combination of efficient charge generation, high detectivity, broad and tunable absorption range, and ease of fabrication on thin and flexible plastic foils make OPDs a very promising technology for conventional inorganic photodetector substitution. Moreover, the ability to use scalable fabrication techniques as well as organic materials’ biocompatibility open up a variety of applications for OPDs in next-generation wearable electronics, such as sensors for the continuous monitoring of health signals and imaging.

## Figures and Tables

**Table 1:** A summary of recent progress made in the development of Flexible OPDs.

Ref	Active Materials	Substrate	Device Type	Flexibility	Responsivity	Detectivity	Response Time
[36]	Tin-based perovskite/PEDOT:PSS	PI	PT	Rb = 8 mm 300 times	∼8.7 × 10^5^ A/W	-	-
[37]	P3HT:PC70BM	PET	PM	1000 bending at R = 8.54 mm	388.4 A/W	-	210 ms
[38]	PCDTBT:PCBM	PI	Photodiode	-	0.21 A/W	3 × 10^12^ Jones	-
[39]	PBTB	PET	PC	2000 bending at theta = 120 degree	0.9 A/W	1.8 × 10^10^ Jones	900 ms
[40]	TQ1:PNDI-T10	PET	Photodiode	-	0.17 A/W	10^11^ Jones	-
[41]	D18:Y6 and D18/Y6	PET	Photodiode	Bending with R = 11 mm	0.35 A/W	9.39 × 10^11^ Jones	0.013 ms
[42]	P3HT:o-IDTBR	PET	Photodiode	1000 bending at R = 1 cm	-	-	-
[43]	PBDTTT-OFT:IEICO-4F	PI	Photodiode	1000 bending at R = 25 micrometer	11.9 A/W	1.7 × 10^15^ Jones	0.037 s
[44]	PDPP-DTT:PC61 BM/cross-linked DPP-DTT	PI	PT	Bending with R = 6 mm	150 A/W	-	0.15 s
[45]	D18	PET	PT	2000 bending at R = 10 mm	10 A/W	1.7 × 10^13^ Jones	few milliseconds
[46]	In_2_O_3_/PTPBT-ET	PI	PT	1000 bending with R = 5 mm	20 A/W	1.4 × 10^12^ Jones	5 ms
[47]	SQ-H:PC61BM	PET	Photodiode	-	0.2 A/W	9 × 10^10^ Jones	-
[48]	MAPbI3 MWs covered by an assembled P3HT	PET	Photodiode	100 bending with theta = 90 degree	400 A/W	-	12 ms
[49]	SEBS: P3HT: ICBA	Silicone elastomer PDMS	Photodiode	-	0.006 A/W	2.3 × 10^10^ Jones	0.06 ms
[50]	P3HT:PCBM/3DG	PET	Photodiode	100 bending with R = 5 mm	5.8 × 10^5^ A/W	3 × 10^15^ Jones	24 ms
[51]	PTB7-Th:COTIC-4F	PEN	Photodiode	100 bending with R = 7.5 mm	≈0.42/A/W	1.25 × 10^13^ Jones	0.021 ms
[52]	PEDOT:PSS/SnSe_2_	ITO/PET	Photodiode	3000 bending	0.73 A/W	-	16 ms
[53]	ZnO/P3HT	PET	Photodiode	-	156 µA/W	0.74 × 10^9^ Jones	40 ms
[54]	RNA	PET	Photodiode	Bending with theta = 150 degree	14.99 µA/W	5.23 × 10^7^ Jones	5 s

## Data Availability

The data presented in this study are available on request from the corresponding author.

## References

[1] Lv C., Hu C., Luo J., Liu S., Qiao Y., Zhang Z., Song J., Shi Y., Cai J., Watanabe A. (2019). Recent Advances in Graphene-Based Humidity Sensors. Nanomaterials.

[2] Dastgeer G., Shahzad Z.M., Chae H., Kim Y.H., Ko B.M., Eom J. (2022). Bipolar Junction Transistor Exhibiting Excellent Output Characteristics with a Prompt Response against the Selective Protein. Adv. Funct. Mater..

[3] Dastgeer G., Afzal A.M., Aziz J., Hussain S., Jaffery S.H.A., Kim D.K., Imran M., Assiri M.A. (2021). Flexible Memory Device Composed of Metal-Oxide and Two-Dimensional Material (SnO2/WTe2) Exhibiting Stable Resistive Switching. Materials.

[4] Nabavi S., Bhadra S. Flexible Inductive Wireless Power Transfer System with an Onboard Temperature Sensor. Proceedings of the 2021 IEEE International Conference on Flexible and Printable Sensors and Systems (FLEPS).

[5] Debbarma S., Bhadra S. (2022). A Flexible Wearable Electrooculogram System with Motion Artifacts Sensing and Reduction. IEEE Trans. Biomed. Circuits Syst..

[6] Nabavi S., Chen Y., Lasry N., Bhadra S. Fully Flexible Organic LED Fabricated by a Solution-based Process. Proceedings of the 2022 IEEE International Conference on Flexible and Printable Sensors and Systems (FLEPS).

[7] Song Y.M., Xie Y., Malyarchuk V., Xiao J., Jung I., Choi K.-J., Liu Z., Park H., Lu C., Kim R.-H. (2013). Digital cameras with designs inspired by the arthropod eye. Nature.

[8] Kim D.-H., Viventi J., Amsden J.J., Xiao J., Vigeland L., Kim Y.-S., Blanco J.A., Panilaitis B., Frechette E.S., Contreras D. (2010). Dissolvable films of silk fibroin for ultrathin conformal bio-integrated electronics. Nat. Mater..

[9] Yokota T., Zalar P., Kaltenbrunner M., Jinno H., Matsuhisa N., Kitanosako H., Tachibana Y., Yukita W., Koizumi M., Someya T. (2016). Ultraflexible organic photonic skin. Sci. Adv..

[10] Jinno H., Fukuda K., Xu X., Park S., Suzuki Y., Koizumi M., Yokota T., Osaka I., Takimiya K., Someya T. (2017). Stretchable and waterproof elastomer-coated organic photovoltaics for washable electronic textile applications. Nat. Energy.

[11] Kim J., Kwon S.-M., Kang Y.K., Kim Y.-H., Lee M.-J., Han K., Facchetti A., Kim M.-G., Park S.K. (2019). A skin-like two-dimensionally pixelized full-color quantum dot photodetector. Sci. Adv..

[12] Konstantatos G., Howard I., Fischer A., Hoogland S., Clifford J.P., Klem E.J.D., Levina L., Sargent E.H. (2006). Ultrasensitive solution-cast quantum dot photodetectors. Nature.

[13] Yang J., Choi M.K., Yang U.J., Kim S.Y., Kim Y.S., Kim J.H., Kim D.-H., Hyeon T. (2020). Toward Full-Color Electroluminescent Quantum Dot Displays. Nano Lett..

[14] Gu L., Poddar S., Lin Y., Long Z., Zhang D., Zhang Q., Shu L., Qiu X., Kam M., Javey A. (2020). A biomimetic eye with a hemispherical perovskite nanowire array retina. Nature.

[15] Peng Z.-Y., Xu J.-L., Zhang J.-Y., Gao X., Wang S.-D. (2018). Solution-Processed High-Performance Hybrid Photodetectors Enhanced by Perovskite/MoS_2_ Bulk Heterojunction. Adv. Mater. Interfaces.

[16] Lee W., Lee J., Yun H., Kim J., Park J., Choi C., Kim D.C., Seo H., Lee H., Yu J.W. (2017). High-resolution spin-on-patterning of perovskite thin films for a multiplexed image sensor array. Adv. Mater..

[17] Khan M.F., Ahmed F., Rehman S., Akhtar I., Rehman M.A., Shinde P.A., Khan K., Kim D.-K., Eom J., Lipsanen H. (2020). High performance complementary WS_2_ devices with hybrid Gr/Ni contacts. Nanoscale.

[18] Sun Z., Yu P., Wang F., Wang F., Yao Y., Zhan X., Wang Z., He J. (2022). High-performance ultraviolet photodetectors based on 2D layered In4/3P2Se6 nanoflakes. Appl. Phys. Lett..

[19] Choi S., Lee H., Ghaffari R., Hyeon T., Kim D.-H. (2016). Recent Advances in Flexible and Stretchable Bio-Electronic Devices Integrated with Nanomaterials. Adv. Mater..

[20] Lan Z., Lee M.-H., Zhu F. (2021). Recent Advances in Solution-Processable Organic Photodetectors and Applications in Flexible Electronics. Adv. Intell. Syst..

[21] Dong T., Simões J., Yang Z. (2020). Flexible photodetector based on 2D materials: Processing, architectures, and applications. Adv. Mater. Interfaces.

[22] Chow P.C.Y., Someya T. (2019). Organic Photodetectors for Next-Generation Wearable Electronics. Adv. Mater..

[23] Jang W., Kim B.G., Seo S., Shawky A., Kim M.S., Kim K., Mikladal B., Kauppinen E.I., Maruyama S., Jeon I. (2021). Strong dark current suppression in flexible organic photodetectors by carbon nanotube transparent electrodes. Nano Today.

[24] Song J.-K., Kim M.S., Yoo S., Koo J.H., Kim D.-H. (2021). Materials and devices for flexible and stretchable photodetectors and light-emitting diodes. Nano Res..

[25] Ren H., Chen J., Li Y., Tang J. (2020). Recent Progress in Organic Photodetectors and their Applications. Adv. Sci..

[26] Brédas J.-L., Norton J.E., Cornil J., Coropceanu V. (2009). Molecular Understanding of Organic Solar Cells: The Challenges. Accounts Chem. Res..

[27] Yu G., Gao J., Hummelen J.C., Wudl F., Heeger A.J. (1995). Polymer Photovoltaic Cells: Enhanced Efficiencies via a Network of Internal Donor-Acceptor Heterojunctions. Science.

[28] Halls J.J.M., Walsh C.A., Greenham N.C., Marseglia E.A., Friend R.H., Moratti S.C., Holmes A.B. (1995). Efficient photodiodes from interpenetrating polymer networks. Nature.

[29] Chow P.C.Y., Bayliss S.L., Lakhwani G., Greenham N.C., Friend R.H. (2015). In Situ Optical Measurement of Charge Transport Dynamics in Organic Photovoltaics. Nano Lett..

[30] Torsi L., Magliulo M., Manoli K., Palazzo G. (2013). Organic field-effect transistor sensors: A tutorial review. Chem. Soc. Rev..

[31] Vandewal K. (2016). Interfacial Charge Transfer States in Condensed Phase Systems. Annu. Rev. Phys. Chem..

[32] Vandewal K., Ma Z., Bergqvist J., Tang Z., Wang E., Henriksson P., Tvingstedt K., Andersson M.R., Zhang F., Inganäs O. (2012). Quantification of Quantum Efficiency and Energy Losses in Low Bandgap Polymer:Fullerene Solar Cells with High Open-Circuit Voltage. Adv. Funct. Mater..

[33] Cnops K., Rand B., Cheyns D., Verreet B., Empl M.A., Heremans P. (2014). 8.4% efficient fullerene-free organic solar cells exploiting long-range exciton energy transfer. Nat. Commun..

[34] Kuribara K., Wang H., Uchiyama N., Fukuda K., Yokota T., Zschieschang U., Jaye C., Fischer D.A., Klauk H., Yamamoto T. (2012). Organic transistors with high thermal stability for medical applications. Nat. Commun..

[35] Zhang G., Zhao J., Chow P.C.Y., Jiang K., Zhang J., Zhu Z., Zhang J., Huang F., Yan H. (2018). Nonfullerene Acceptor Molecules for Bulk Heterojunction Organic Solar Cells. Chem. Rev..

[36] Liu C.K., Tai Q., Wang N., Tang G., Hu Z., Yan F. (2020). Lead-Free Perovskite/Organic Semiconductor Vertical Heterojunction for Highly Sensitive Photodetectors. ACS Appl. Mater. Interfaces.

[37] Shi L., Song J., Zhang Y., Li G., Wang W., Hao Y., Wu Y., Cui Y. (2020). High performance flexible organic photomultiplication photodetector based on an ultra-thin silver film transparent electrode. Nanotechnology.

[38] Van Breemen A.J., Simon M., Tousignant O., Shanmugam S., van der Steen J.L., Akkerman H.B., Kronemeijer A., Ruetten W., Raaijmakers R., Alving L. (2020). Curved digital X-ray detectors. npj Flex. Electron..

[39] Lv L., Dang W., Wu X., Chen H., Wang T., Qin L., Wei Z., Zhang K., Shen G., Huang H. (2020). Flexible Short-Wave Infrared Image Sensors Enabled by High-Performance Polymeric Photodetectors. Macromolecules.

[40] Xia Y., Aguirre L.E., Xu X., Inganäs O. (2020). All-Polymer High-Performance Photodetector through Lamination. Adv. Electron. Mater..

[41] Wei Y., Chen H., Liu T., Wang S., Jiang Y., Song Y., Zhang J., Zhang X., Lu G., Huang F. (2021). Self-Powered Organic Photodetectors with High Detectivity for Near Infrared Light Detection Enabled by Dark Current Reduction. Adv. Funct. Mater..

[42] Large M.J., Posar J.A., Mozer A.J., Nattestad A., Alnaghy S., Carolan M., Sellin P.J., Davies J., Pastuovic Z., Lerch M.L.F. (2021). Flexible Polymer X-ray Detectors with Non-fullerene Acceptors for Enhanced Stability: Toward Printable Tissue Equivalent Devices for Medical Applications. ACS Appl. Mater. Interfaces.

[43] Jiang Z., Yu K., Wang H., Rich S., Yokota T., Fukuda K., Someya T. (2020). Ultraflexible Integrated Organic Electronics for Ultrasensitive Photodetection. Adv. Mater. Technol..

[44] Li Q., Ran Y., Shi W., Qin M., Sun Y., Kuang J., Wang H., Chen H., Guo Y., Liu Y. (2020). High-performance near-infrared polymeric phototransistors realized by combining cross-linked polymeric semiconductors and bulk heterojunction bilayer structures. Appl. Mater. Today.

[45] Huang H., Jiang L., Peng J., Qi Y., Bai S., Lin Q. (2021). High-Performance Organic Phototransistors Based on D18, a High-Mobility and Unipolar Polymer. Chem. Mater..

[46] Li D., Du J., Tang Y., Liang K., Wang Y., Ren H., Wang R., Meng L., Zhu B., Li Y. (2021). Flexible and Air-Stable Near-Infrared Sensors Based on Solution-Processed Inorganic–Organic Hybrid Phototransistors. Adv. Funct. Mater..

[47] Kim J.H., Liess A., Stolte M., Krause A., Stepanenko V., Zhong C., Bialas D., Spano F., Würthner F. (2021). An Efficient Narrowband Near-Infrared at 1040 nm Organic Photodetector Realized by Intermolecular Charge Transfer Mediated Coupling Based on a Squaraine Dye. Adv. Mater..

[48] Zhang J., Zhong W., Liu Y., Huang J., Deng S., Zhang M., Kwok H., Li G. (2021). A High-Performance Photodetector Based on 1D Perovskite Radial Heterostructure. Adv. Opt. Mater..

[49] Park Y., Fuentes-Hernandez C., Kim K., Chou W.-F., Larrain F.A., Graham S., Pierron O.N., Kippelen B. (2021). Skin-like low-noise elastomeric organic photodiodes. Sci. Adv..

[50] Ge Z., Xu N., Zhu Y., Zhao K., Ma Y., Li G., Chen Y. (2022). Visible to Mid-Infrared Photodetection Based on Flexible 3D Graphene/Organic Hybrid Photodetector with Ultrahigh Responsivity at Ambient Conditions. ACS Photon..

[51] Simões J., Dong T., Yang Z. (2022). Non-Fullerene Acceptor Organic Photodetector for Skin-Conformable Photoplethysmography Applications. Adv. Mater. Interfaces.

[52] Reddy B.K.S., Veeralingam S., Borse P.H., Badhulika S. (2022). A flexible, rapid response, hybrid inorganic–organic SnSe 2 –PEDOT:PSS bulk heterojunction based high-performance broadband photodetector. Mater. Chem. Front..

[53] Du X., Tian W., Pan J., Hui B., Sun J., Zhang K., Xia Y. (2021). Piezo-phototronic effect promoted carrier separation in coaxial p-n junctions for self-powered photodetector. Nano Energy.

[54] Hu C., Chen H., Li L., Huang H., Shen G. (2022). Ti3C2Tx MXene-RAN van der Waals Heterostructure-Based Flexible Transparent NIR Photodetector Array for 1024 Pixel Image Sensing Application. Adv. Mater. Technol..

[55] Munzenrieder N., Zysset C., Kinkeldei T., Troster G. (2012). Design Rules for IGZO Logic Gates on Plastic Foil Enabling Operation at Bending Radii of 3.5 mm. IEEE Trans. Electron Devices.

[56] Takei K., Takahashi T., Ho J.C., Ko H., Gillies A.G., Leu P.W., Fearing R.S., Javey A. (2010). Nanowire active-matrix circuitry for low-voltage macroscale artificial skin. Nat. Mater..

[57] Liu X., Wang C., Cai B., Xiao X., Guo S., Fan Z., Li J., Duan X., Liao L. (2012). Rational Design of Amorphous Indium Zinc Oxide/Carbon Nanotube Hybrid Film for Unique Performance Transistors. Nano Lett..

[58] Wang C., Chien J.-C., Takei K., Takahashi T., Nah J., Niknejad A.M., Javey A. (2012). Extremely Bendable, High-Performance Integrated Circuits Using Semiconducting Carbon Nanotube Networks for Digital, Analog, and Radio-Frequency Applications. Nano Lett..

[59] Yi H.T., Payne M.M., Anthony J., Podzorov V. (2012). Ultra-flexible solution-processed organic field-effect transistors. Nat. Commun..

[60] Sekitani T., Zschieschang U., Klauk H., Someya T. (2010). Flexible organic transistors and circuits with extreme bending stability. Nat. Mater..

[61] Zhang L., Wang H., Zhao Y., Guo Y., Hu W., Yu G., Liu Y. (2013). Substrate-Free Ultra-Flexible Organic Field-Effect Transistors and Five-Stage Ring Oscillators. Adv. Mater..

[62] Khodagholy D., Rivnay J., Sessolo M., Gurfinkel M., Leleux P., Jimison L.H., Stavrinidou E., Herve T., Sanaur S., Owens R. (2013). High transconductance organic electrochemical transistors. Nat. Commun..

[63] Kim D.-H., Ahn J.-H., Choi W.M., Kim H.-S., Kim T.-H., Song J., Huang Y.Y., Liu Z., Lu C., Rogers J.A. (2008). Stretchable and Foldable Silicon Integrated Circuits. Nature.

[64] Kaltenbrunner M., White M., Głowacki E.D., Sekitani T., Someya T., Sariciftci N.S., Bauer S. (2012). Ultrathin and lightweight organic solar cells with high flexibility. Nat. Commun..

[65] Kaltenbrunner M., Sekitani T., Reeder J., Yokota T., Kuribara K., Tokuhara T., Drack M., Schwödiauer R., Graz I., Bauer-Gogonea S. (2013). An ultra-lightweight design for imperceptible plastic electronics. Nature.

[66] Khodagholy D., Doublet T., Quilichini P., Gurfinkel M., LeLeux P., Ghestem A., Ismailova E., Hervé T., Sanaur S., Bernard C. (2013). In vivo recordings of brain activity using organic transistors. Nat. Commun..

[67] Jiang D.-H., Liao Y.-C., Cho C.-J., Veeramuthu L., Liang F.-C., Wang T.-C., Chueh C.-C., Satoh T., Tung S.-H., Kuo C.-C. (2020). Facile Fabrication of Stretchable Touch-Responsive Perovskite Light-Emitting Diodes Using Robust Stretchable Composite Electrodes. ACS Appl. Mater. Interfaces.

[68] Shin M., Song J.H., Lim G.-H., Lim B., Park J.-J., Jeong U. (2014). Highly Stretchable Polymer Transistors Consisting Entirely of Stretchable Device Components. Adv. Mater..

[69] Wang S., Xu J., Wang W., Wang G.-J.N., Rastak R., Molina-Lopez F., Chung J.W., Niu S., Feig V.R., Lopez J. (2018). Skin electronics from scalable fabrication of an intrinsically stretchable transistor array. Nature.

[70] Lee P., Lee J., Lee H., Yeo J., Hong S., Nam K.H., Lee D., Lee S.S., Ko S.H. (2012). Highly Stretchable and Highly Conductive Metal Electrode by Very Long Metal Nanowire Percolation Network. Adv. Mater..

[71] Kim T.-H., Lee C.-S., Kim S., Hur J., Lee S., Shin K.W., Yoon Y.-Z., Choi M.K., Yang J., Kim D.-H. (2017). Fully Stretchable Optoelectronic Sensors Based on Colloidal Quantum Dots for Sensing Photoplethysmographic Signals. ACS Nano.

[72] Jinno H., Yokota T., Koizumi M., Yukita W., Saito M., Osaka I., Fukuda K., Someya T. (2021). Self-powered ultraflexible photonic skin for continuous bio-signal detection via air-operation-stable polymer light-emitting diodes. Nat. Commun..

[73] Jeon I., Xiang R., Shawky A., Matsuo Y., Maruyama S. (2018). Single-Walled Carbon Nanotubes in Emerging Solar Cells: Synthesis and Electrode Applications. Adv. Energy Mater..

[74] He W., Ye C. (2015). Flexible Transparent Conductive Films on the Basis of Ag Nanowires: Design and Applications: A Review. J. Mater. Sci. Technol..

[75] Huang J., Wang X., Kim Y., Demello A.J., Bradley D.D.C., Demello J.C. (2006). High efficiency flexible ITO-free polymer/fullerene photodiodes. Phys. Chem. Chem. Phys..

[76] Du J., Pei S., Ma L., Cheng H.-M. (2014). 25th Anniversary Article: Carbon Nanotube- and Graphene-Based Transparent Conductive Films for Optoelectronic Devices. Adv. Mater..

[77] Mei X., Ouyang J. (2011). Highly conductive and transparent single-walled carbon nanotube thin films fabricated by gel coating. J. Mater. Chem..

[78] Cesarini M., Brigante B., Caironi M., Natali D. (2018). Reproducible, High Performance Fully Printed Photodiodes on Flexible Substrates through the Use of a Polyethylenimine Interlayer. ACS Appl. Mater. Interfaces.

[79] Hu L., Kim H.S., Lee J.-Y., Peumans P., Cui Y. (2010). Scalable coating and properties of transparent, flexible, silver nanowire electrodes. ACS Nano..

[80] Lee J.-Y., Connor S.T., Cui A.Y., Peumans P. (2008). Solution-Processed Metal Nanowire Mesh Transparent Electrodes. Nano Lett..

[81] Song M., You D.S., Lim K., Park S., Jung S., Kim C.S., Kim D.-H., Kim D.-G., Kim J.-K., Park J. (2013). Highly Efficient and Bendable Organic Solar Cells with Solution-Processed Silver Nanowire Electrodes. Adv. Funct. Mater..

[82] Hu W., Huang W., Yang S., Wang X., Jiang Z., Zhu X., Zhou H., Liu H., Zhang Q., Zhuang X. (2017). High-Performance Flexible Photodetectors based on High-Quality Perovskite Thin Films by a Vapor-Solution Method. Adv. Mater..

[83] Yu Z., Zhang Q., Li L., Chen Q., Niu X., Liu J., Pei Q. (2010). Highly Flexible Silver Nanowire Electrodes for Shape-Memory Polymer Light-Emitting Diodes. Adv. Mater..

[84] Gaynor W., Burkhard G.F., McGehee M.D., Peumans P. (2011). Smooth Nanowire/Polymer Composite Transparent Electrodes. Adv. Mater..

[85] Sun K., Zhang S., Li P., Xia Y., Zhang X., Du D., Isikgor F.H., Ouyang J. (2015). Review on application of PEDOTs and PEDOT:PSS in energy conversion and storage devices. J. Mater. Sci. Mater. Electron..

[86] Ouyang S., Xie Y., Wang D., Zhu D., Xu X., Tan T., Fong H.H. (2015). Surface Patterning of PEDOT:PSS by Photolithography for Organic Electronic Devices. J. Nanomater..

[87] Fischer R., Gregori A., Sahakalkan S., Hartmann D., Büchele P., Tedde S.F., Schmidt O. (2018). Stable and highly conductive carbon nanotube enhanced PEDOT:PSS as transparent electrode for flexible electronics. Org. Electron..

[88] Zhou Y., Fuentes-Hernandez C., Shim J., Meyer J., Giordano A.J., Li H., Winget P., Papadopoulos T., Cheun H., Kim J. (2012). A Universal Method to Produce Low–Work Function Electrodes for Organic Electronics. Science.

[89] Li Z., Qin F., Liu T., Ge R., Meng W., Tong J., Xiong S., Zhou Y. (2015). Optical properties and conductivity of PEDOT:PSS films treated by polyethylenimine solution for organic solar cells. Org. Electron..

[90] Xu X., Sun L., Shen K., Zhang S. (2019). Organic and hybrid organic-inorganic flexible optoelectronics: Recent advances and perspectives. Synth. Met..

[91] Dimitrov S.D., Durrant J.R. (2013). Materials Design Considerations for Charge Generation in Organic Solar Cells. Chem. Mater..

[92] Fan H., Zhu X. (2015). Development of small-molecule materials for high-performance organic solar cells. Sci. China Ser. B Chem..

[93] Wei Y., Yu J., Qin L., Chen H., Wu X., Wei Z., Zhang X., Xiao Z., Ding L., Gao F. (2021). A universal method for constructing high efficiency organic solar cells with stacked structures. Energy Environ. Sci..

[94] Fuentes-Hernandez C., Chou W.F., Khan T.M., Diniz L., Lukens J., Larrain F.A., Kippelen B. (2020). Large-area low-noise flexible organic photodiodes for detecting faint visible light. Science.

[95] Gao Y., Yi Y., Wang X., Meng H., Lei D., Yu X., Chu P.K., Li J. (2019). A Novel Hybrid-Layered Organic Phototransistor Enables Efficient Intermolecular Charge Transfer and Carrier Transport for Ultrasensitive Photodetection. Adv. Mater..

[96] Lei Y., Mao L., Zhu H., Yao J. (2021). Development of catechol-functionalized chitosan/poly(vinyl alcohol) nanocomposite films incorporated with dual network coated layered clay for active packaging applications. J. Appl. Polym. Sci..

[97] Peng Y., Lv W., Yao B., Fan G., Chen D., Gao P., Zhou M., Wang Y. (2013). High performance near infrared photosensitive organic field-effect transistors realized by an organic hybrid planar-bulk heterojunction. Org. Electron..

[98] Rim Y.S., Ok K.-C., Yang Y.M., Chen H., Bae S.-H., Wang C., Huang Y., Park J.-S. (2016). Boosting Responsivity of Organic–Metal Oxynitride Hybrid Heterointerface Phototransistor. ACS Appl. Mater. Interfaces.

[99] Anthony J.E., Facchetti A., Heeney M., Marder S.R., Zhan X. (2010). n-Type Organic Semiconductors in Organic Electronics. Adv. Mater..

[100] Cortizo-Lacalle D., Gozalvez C., Olano M., Sun X., Melle-Franco M., Hueso L.E., Mateo-Alonso A. (2015). Bisthiadiazole-fused tetraazapentacenequinone: An air-stable solution-processable n-type organic semiconductor. Org. Lett..

[101] Kalita A., Subbarao N.V.V., Iyer P.K. (2015). Large-Scale Molecular Packing and Morphology-Dependent High Performance Organic Field-Effect Transistor by Symmetrical Naphthalene Diimide Appended with Methyl Cyclohexane. J. Phys. Chem. C.

[102] Lee M.Y., Park J., Oh J.H. (2019). High-Performance Ambipolar Organic Phototransistors Based on Core–Shell p–n Junction Organic Single Crystals. ACS Appl. Electron. Mater..

[103] Kim S., Lim T., Sim K., Kim H., Choi Y., Park K., Pyo S. (2011). Light Sensing in a Photoresponsive, Organic-Based Complementary Inverter. ACS Appl. Mater. Interfaces.

[104] Nam S., Seo J., Han H., Kim H., Bradley D.D.C., Kim Y. (2017). Efficient Deep Red Light-Sensing All-Polymer Phototransistors with *p*-type/*n*-type Conjugated Polymer Bulk Heterojunction Layers. ACS Appl. Mater. Interfaces.

[105] Liu M., Wang H., Tang Q., Zhao X., Tong Y., Liu Y. (2018). Ultrathin Air-Stable n-Type Organic Phototransistor Array for Conformal Optoelectronics. Sci. Rep..

[106] Guo D., Yang L., Zhao J., Li J., He G., Yang D., Wang L., Vadim A., Ma D. (2021). Visible-blind ultraviolet narrowband photomultiplication-type organic photodetector with an ultrahigh external quantum efficiency of over 1,000,000%. Mater. Horizons.

[107] Khan Y., Ostfeld A.E., Lochner C.M., Pierre A., Arias A.C. (2016). Monitoring of Vital Signs with Flexible and Wearable Medical Devices. Adv. Mater..

[108] Park S., Fukuda K., Wang M., Lee C., Yokota T., Jin H., Jinno H., Kimura H., Zalar P., Matsuhisa N. (2018). Ultraflexible Near-Infrared Organic Photodetectors for Conformal Photoplethysmogram Sensors. Adv. Mater..

[109] Gogurla N., Roy B., Min K., Park J., Kim S. (2020). A Skin-Inspired, Interactive, and Flexible Optoelectronic Device with Hydrated Melanin Nanoparticles in a Protein Hydrogel–Elastomer Hybrid. Adv. Mater. Technol..

[110] Lee H., Jiang Z., Yokota T., Fukuda K., Park S., Someya T. (2021). Stretchable organic optoelectronic devices: Design of materials, structures, and applications. Mater. Sci. Eng. R Rep..

[111] Kim R.-H., Kim D., Xiao J., Kim B.H., Park S.-I., Panilaitis B., Ghaffari R., Yao J., Li M., Liu Z. (2010). Waterproof AlInGaP optoelectronics on stretchable substrates with applications in biomedicine and robotics. Nat. Mater..

[112] Lee Y., Oh J.Y., Xu W., Kim O., Kim T.R., Kang J., Kim Y., Son D., Tok J.B.-H., Park M.J. (2018). Stretchable organic optoelectronic sensorimotor synapse. Sci. Adv..

[113] Ko H.C., Stoykovich M.P., Song J., Malyarchuk V., Choi W.M., Yu C.-J., Iii J.B.G., Xiao J., Wang S., Huang Y. (2008). A hemispherical electronic eye camera based on compressible silicon optoelectronics. Nature.

[114] Peng W., Wang L., Murali B., Ho K.-T., Bera A., Cho N., Kang C.-F., Burlakov V.M., Pan J., Sinatra L. (2016). Solution-Grown Monocrystalline Hybrid Perovskite Films for Hole-Transporter-Free Solar Cells. Adv. Mater..

[115] Peng W., Yin J., Ho K.T., Ouellette O., De Bastiani M., Murali B., El Tall O., Shen C., Miao X., Pan J. (2017). Ultralow Self-Doping in Two-dimensional Hybrid Perovskite Single Crystals. Nano Lett..

[116] Lee M.M., Teuscher J., Miyasaka T., Murakami T.N., Snaith H.J. (2012). Efficient Hybrid Solar Cells Based on Meso-Superstructured Organometal Halide Perovskites. Science.

[117] Stranks S.D., Eperon G.E., Grancini G., Menelaou C., Alcocer M.J.P., Leijtens T., Herz L.M., Petrozza A., Snaith H.J. (2013). Electron-Hole Diffusion Lengths Exceeding 1 Micrometer in an Organometal Trihalide Perovskite Absorber. Science.

[118] Hu X., Zhang X., Liang L., Bao J., Li S., Yang W., Xie Y. (2014). High-Performance Flexible Broadband Photodetector Based on Organolead Halide Perovskite. Adv. Funct. Mater..

[119] Qian L., Sun Y., Wu M., Li C., Xie D., Ding L., Shi G. (2018). A lead-free two-dimensional perovskite for a high-performance flexible photoconductor and a light-stimulated synaptic device. Nanoscale.

[120] Song J., Xu L., Li J., Xue J., Dong Y., Li X., Zeng H. (2016). Monolayer and Few-Layer All-Inorganic Perovskites as a New Family of Two-Dimensional Semiconductors for Printable Optoelectronic Devices. Adv. Mater..

[121] Xie C., You P., Liu Z., Li L., Yan F. (2017). Ultrasensitive broadband phototransistors based on perovskite/organic-semiconductor vertical heterojunctions. Light. Sci. Appl..

[122] Liu Y., Zhang Y., Zhu X., Feng J., Spanopoulos I., Ke W., Liu S. (2021). Triple-Cation and Mixed-Halide Perovskite Single Crystal for High-Performance X-ray Imaging. Adv. Mater..

[123] Jing H., Peng R., Ma R.-M., He J., Zhou Y., Yang Z., Li C.-Y., Liu Y., Guo X., Zhu Y. (2020). Flexible Ultrathin Single-Crystalline Perovskite Photodetector. Nano Lett..

[124] Nie W., Tsai H., Asadpour R., Blancon J.-C., Neukirch A.J., Gupta G., Crochet J.J., Chhowalla M., Tretiak S., Alam M.A. (2015). High-efficiency solution-processed perovskite solar cells with millimeter-scale grains. Science.

[125] Alsalloum A.Y., Turedi B., Almasabi K., Zheng X., Naphade R., Stranks S.D., Mohammed O.F., Bakr O.M. (2021). 22.8%-Efficient single-crystal mixed-cation inverted perovskite solar cells with a near-optimal bandgap. Energy Environ. Sci..

[126] Wang H., Li S., Liu X., Shi Z., Fang X., He J. (2021). Low-dimensional metal halide perovskite photodetectors. Adv. Mater..

[127] Yang Y., Chen H., Hu C., Yang S. (2019). Polyethyleneimine-functionalized carbon nanotubes as an interlayer to bridge perovskite/carbon for all inorganic carbon-based perovskite solar cells. J. Mater. Chem. A.

[128] Li F., Wang H., Kufer D., Liang L., Yu W., Alarousu E., Wu T. (2017). Ultrahigh Carrier Mobility Achieved in Photoresponsive Hybrid Perovskite Films via Coupling with Single-Walled Carbon Nanotubes. Adv. Mater..

[129] Wu H., Si H., Zhang Z., Kang Z., Wu P., Zhou L., Zhang S., Zhang Z., Liao Q., Zhang Y. (2018). All-Inorganic Perovskite Quantum Dot-Monolayer MoS_2_ Mixed-Dimensional van der Waals Heterostructure for Ultrasensitive Photodetector. Adv. Sci..

[130] Zhang X., Zheng E., Esopi M.R., Cai C., Yu Q. (2018). Flexible Narrowband Ultraviolet Photodetectors with Photomultiplication Based on Wide Band Gap Conjugated Polymer and Inorganic Nanoparticles. ACS Appl. Mater. Interfaces.

[131] Hansen M.P., Malchow D.S. (2008). Overview of SWIR detectors, cameras, and applications. THERMOSENSE XXX.

[132] Fallon K.J., Wijeyasinghe N., Yaacobi-Gross N., Ashraf R.S., Freeman D.M.E., Palgrave R.G., Al-Hashimi M., Marks T.J., McCulloch I., Anthopoulos T.D. (2015). A Nature-Inspired Conjugated Polymer for High Performance Transistors and Solar Cells. Macromolecules.

[133] Fallon K., Wijeyasinghe N., Manley E., Marks T., Anthopoulos T.D., Bronstein H.A. (2017). Indolo-naphthyridine-6,13-dione thiophene building block for conjugated polymer electronics: Molecular origin of ultrahigh n-type mobility (Conference Presentation). Organic Field-Effect Transistors XVI.

[134] Chang J., Sonar P., Lin Z., Zhang C., Zhang J., Hao Y., Wu J. (2016). Controlling aggregation and crystallization of solution processed diketopyrrolopyrrole based polymer for high performance thin film transistors by pre-metered slot die coating process. Org. Electron..

[135] Etxebarria I., Ajuria J., Pacios R. (2015). Polymer:fullerene solar cells: Materials, processing issues, and cell layouts to reach power conversion efficiency over 10%, a review. J. Photon- Energy.

[136] Wang F., Han Y., Lim C.S., Lu Y., Wang J., Xu J., Chen H., Zhang C., Hong M., Liu X. (2010). Simultaneous phase and size control of upconversion nanocrystals through lanthanide doping. Nature.

[137] Ghosh P., Patra A. (2008). Tuning of Crystal Phase and Luminescence Properties of Eu^3+^ Doped Sodium Yttrium Fluoride Nanocrystals. J. Phys. Chem. C.

[138] Zhang L., Zhu Y.J. (2009). Microwave Hydrothermal Synthesis of Hexagonal NaYF4 and Yb3+, Er3+-doped NaYF4 Microtubes. J. Inorg. Mater..

[139] Auzel F. (2003). Upconversion and Anti-Stokes Processes with f and d Ions in Solids. Chem. Rev..

[140] Zhang X., Yang S., Zhou H., Liang J., Liu H., Xia H., Zhu X., Jiang Y., Zhang Q., Hu W. (2017). Perovskite-Erbium Silicate Nanosheet Hybrid Waveguide Photodetectors at the Near-Infrared Telecommunication Band. Adv. Mater..

[141] Xiang H., Hu Z., Billot L., Aigouy L., Zhang W., McCulloch I., Chen Z. (2019). Heavy-Metal-Free Flexible Hybrid Polymer-Nanocrystal Photodetectors Sensitive to 1.5 μm Wavelength. ACS Appl. Mater. Interfaces.

[142] Kufer D., Nikitskiy I., Lasanta T., Navickaite G., Koppens F.H.L., Konstantatos G. (2015). Hybrid 2D-0D MoS2-PbS Quantum Dot Photodetectors. Adv. Mater..

[143] Wang X., Wang P., Wang J., Hu W., Zhou X., Guo N., Huang H., Sun S., Shen H., Lin T. (2015). Ultrasensitive and Broadband MoS_2_Photodetector Driven by Ferroelectrics. Adv. Mater..

[144] Liu W., Lee J.-S., Talapin D.V. (2012). III–V Nanocrystals Capped with Molecular Metal Chalcogenide Ligands: High Electron Mobility and Ambipolar Photoresponse. J. Am. Chem. Soc..

[145] Zhao X., Song L., Zhao R., Tan M.C. (2018). High-Performance and Flexible Shortwave Infrared Photodetectors Using Composites of Rare Earth-Doped Nanoparticles. ACS Appl. Mater. Interfaces.

[146] Renner R., Stolte M., Heitmüller J., Brixner T., Lambert C., Würthner F. (2021). Substituent-dependent absorption and fluorescence properties of perylene bisimide radical anions and dianions. Mater. Horizons.

[147] Zhang Y., Feng Q., Hao R., Zhang M. (2022). Fabrication of Large-Area Short-Wave Infrared Array Photodetectors under High Operating Temperature by High Quality PtS_2_ Continuous Films. Electronics.

[148] Lu J., Ye Q., Ma C., Zheng Z., Yao J., Yang G. (2022). Dielectric Contrast Tailoring for Polarized Photosensitivity toward Multiplexing Optical Communications and Dynamic Encrypt Technology. ACS Nano.

[149] Saritha K., Reddy A.S., Reddy K.R. (2017). Investigation on Optical Properties of SnSe 2 Thin Films Synthesized by Two–Stage Process. Mater. Today: Proc..

[150] Hsieh D., Xia Y., Wray L., Qian D., Pal A., Dil J.H., Hasan M.Z. (2009). Observation of Unconventional Quantum Spin Textures in Topological Insulators. Science.

[151] Maier L., Bocquillon E., Grimm M., Oostinga J.B., Ames C., Gould C., Molenkamp L.W. (2015). Phase-sensitive SQUIDs based on the 3D topological insulator HgTe. Phys. Scr..

[152] Chen C., Xie Z., Feng Y., Yi H., Liang A., He S., Zhou X.J. (2013). Tunable Dirac Fermion Dynamics in Topological Insulators. Sci. Rep..

[153] Zhang H., Liu C.-X., Qi X.-L., Dai X., Fang Z., Zhang S.-C. (2009). Topological insulators in Bi2Se3, Bi2Te3 and Sb2Te3 with a single Dirac cone on the surface. Nat. Phys..

[154] Zhang H.-J., Chadov S., Müchler L., Yan B., Qi X.-L., Kübler J., Zhang S.-C., Felser C. (2011). Topological Insulators in Ternary Compounds with a Honeycomb Lattice. Phys. Rev. Lett..

[155] Yang M., Wang J., Zhao Y., He L., Ji C., Liu X., Jiang Y. (2019). Three-Dimensional Topological Insulator Bi 2 Te 3/Organic Thin Film Heterojunction Photodetector with Fast and Wideband Response from 450 to 3500 Nanometers. ACS Nano..

[156] Kmiec M.M., Hou H., Kuppusamy M.L., Drews T.M., Prabhat A., Petryakov S.V., Demidenko E., Schaner P.E., Buckey J.C., Blank A. (2018). Transcutaneous oxygen measurement in humans using a paramagnetic skin adhesive film. Magn. Reson. Med..

[157] Ryu G.-S., You J., Kostianovskii V., Lee E.-B., Kim Y., Park C., Noh Y.-Y. (2018). Flexible and Printed PPG Sensors for Estimation of Drowsiness. IEEE Trans. Electron Devices.

[158] Xu H., Liu J., Zhang J., Zhou G., Luo N., Zhao N. (2017). Flexible Organic/Inorganic Hybrid Near-Infrared Photoplethysmogram Sensor for Cardiovascular Monitoring. Adv. Mater..

[159] Yudovsky D., Nouvong A., Schomacker K., Pilon L. (2011). Assessing diabetic foot ulcer development risk with hyperspectral tissue oximetry. J. Biomed. Opt..

[160] Ericson M., Wilson M., Coté G., Baba J., Xu W., Bobrek M., Britton C., Hileman M., Moore M., Emery M. (2004). Implantable sensor for blood flow monitoring after transplant surgery. Minim. Invasive Ther. Allied Technol..

[161] Boas D.A., Franceschini M.A. (2011). Haemoglobin oxygen saturation as a biomarker: The problem and a solution. Philos. Trans. R. Soc. London. Ser. A: Math. Phys. Eng. Sci..

[162] Sen C.K. (2009). Wound healing essentials: Let there be oxygen. Wound Repair Regen..

[163] Khan Y., Han D., Pierre A., Ting J., Wang X., Lochner C.M., Bovo G., Yaacobi-Gross N., Newsome C., Wilson R. (2018). A flexible organic reflectance oximeter array. Proc. Natl. Acad. Sci. USA.

[164] Lee H., Kim E., Lee Y., Kim H., Lee J., Kim M., Yoo S. (2018). Toward all-day wearable health monitoring: An ultralow-power, reflective organic pulse oximetry sensing patch. Sci. Adv..

[165] Babilas P., Lamby P., Prantl L., Schreml S., Jung E.M., Liebsch G., Wolfbeis O.S., Landthaler M., Szeimies R.-M., Abels C. (2008). Transcutaneous pO_2_imaging during tourniquet-induced forearm ischemia using planar optical oxygen sensors. Ski. Res. Technol..

[166] Deckman I., Lechêne P.B., Pierre A., Arias A.C. (2018). All-printed full-color pixel organic photodiode array with a single active layer. Org. Electron..

[167] Theuwissen J.P., IEEE Image processing chain in digital still cameras. Proceedings of the 2004 Symposium on VLSI Circuits. Digest of Technical Papers (IEEE Cat. No.04CH37525).

[168] Zalar P., Matsuhisa N., Suzuki T., Enomoto S., Koizumi M., Yokota T., Sekino M., Someya T. (2018). A Monolithically Processed Rectifying Pixel for High-Resolution Organic Imagers. Adv. Electron. Mater..

[169] Hu H., Larson R.G. (2006). Marangoni effect reverses coffee-ring depositions. J. Phys. Chem. B.

[170] Grau G., Cen J., Kang H., Kitsomboonloha R., Scheideler W.J., Subramanian V. (2016). Gravure-printed electronics: Recent progress in tooling development, understanding of printing physics, and realization of printed devices. Flex. Print. Electron..

[171] Eckstein R., Strobel N., Rödlmeier T., Glaser K., Lemmer U., Hernandez-Sosa G. (2018). Fully Digitally Printed Image Sensor Based on Organic Photodiodes. Adv. Opt. Mater..

[172] Thirimanne H.M., Jayawardena K.D.G.I., Parnell A.J., Bandara R.M.I., Karalasingam A., Pani S., Huerdler J.E., Lidzey D.G., Tedde S.F., Nisbet A. (2018). High sensitivity organic inorganic hybrid X-ray detectors with direct transduction and broadband response. Nat. Commun..

[173] Posar J.A., Large M., Alnaghy S., Paino J.R., Butler D.J., Griffith M.J., Petasecca M. (2021). Towards high spatial resolution tissue-equivalent dosimetry for microbeam rathation therapy using organic semiconductors. J. Synchrotron Radiat..

[174] Posar J.A., Davis J., Alnaghy S., Wilkinson D., Cottam S., Lee D.M., Griffith M.J. (2021). Polymer Photodetectors for Printable, Flexible, and Fully Tissue Equivalent X-Ray Detection with Zero-Bias Operation and Ultrafast Temporal Responses. Adv. Mater. Technol..

[175] Nakamura H., Shirakawa Y., Kitamura H., Yamada T., Shidara Z., Yokozuka T., Nguyen P., Takahashi T., Takahashi S. (2013). Blended polyethylene terephthalate and polyethylene naphthalate polymers for scintillation base substrates. Radiat. Meas..

[176] Xu X., Fukuda K., Karki A., Park S., Kimura H., Jinno H., Watanabe N., Yamamoto S., Shimomura S., Kitazawa D. (2018). Thermally stable, highly efficient, ultraflexible organic photovoltaics. Proc. Natl. Acad. Sci. USA.

[177] Park S., Heo S.W., Lee W., Inoue D., Jiang Z., Yu K., Jinno H., Hashizume D., Sekino M., Yokota T. (2018). Self-powered ultra-flexible electronics via nano-grating-patterned organic photovoltaics. Nature.

